# Nup98 Is Subverted from Annulate Lamellae by Hepatitis C Virus Core Protein to Foster Viral Assembly

**DOI:** 10.1128/mbio.02923-21

**Published:** 2022-03-08

**Authors:** Bertrand Boson, Chloé Mialon, Konstanze Schichl, Solène Denolly, François-Loïc Cosset

**Affiliations:** a CIRI—Centre International de Recherche en Infectiologie, Université Lyon, Université Claude Bernard Lyon 1, Inserm, U1111, CNRS, UMR5308, ENS Lyon, Lyon, France; St. Louis University; Washington University School of Medicine

**Keywords:** hepatitis C virus, annulate lamellae, Nup98, viral RNA, assembly

## Abstract

Nup98, an essential component of the nuclear pore that also participates in annulate lamella pore structures localized in the cytosol, is involved in hepatitis C virus (HCV) assembly. Here, we combined confocal microscopy and biochemical assays to study the interplay between Nup98, core (i.e., the HCV capsid protein), and viral genomes. Our results show that in HCV-infected cells, core protein is necessary and sufficient to induce relocalization of Nup98 from annulate lamellae to lipid droplet-apposed areas in which core/NS5A and HCV genomic RNA [(+)RNA] are clustered to promote viral assembly. Furthermore, we found that Nup98 interacts with HCV RNA and that upon Nup98 downregulation, the viral (+)RNA genome was specifically excluded from areas that contain active translating ribosomes and the core and NS5A proteins. Altogether, these results indicate that Nup98 is recruited by HCV core from annulate lamellae to viral assembly sites to locally increase the concentration of (+)RNA genome, which may favor its encapsidation into nascent virions.

## INTRODUCTION

Hepatitis C virus (HCV) is a positive-strand RNA virus of the *Flaviviridae* family and is a major cause of liver disease worldwide, with an estimated 71 million infected people (1). In recent years, new treatments based on direct-acting antivirals (DAAs) (reviewed in reference 2) have emerged and can now cure most patients; however, there remain major challenges in basic, translational, and clinical research (3).

The HCV genome encodes a single polyprotein that is processed in 10 mature viral proteins consisting of (i) an assembly module (core-E1-E2-p7-NS2) encompassing the capsid protein (core) as well as the E1 and E2 surface glycoproteins that are incorporated in viral particles, as well as the p7 and NS2 proteins that support virion assembly, and (ii) a replication module encompassing the nonstructural proteins NS3, NS4A, NS4B, NS5A, and NS5B, which are sufficient to support viral RNA replication but which also contribute to virion production.

The assembly of HCV particles occurs at endoplasmic reticulum (ER)-derived membranes in close proximity to lipid droplets (LD) and viral replication complexes (4). In addition, significant membrane rearrangements, called the “membranous web” (MW), occur in HCV-infected cells. These membranes originate from the ER and are supposed to form a viral organelle that allows concentration in a protected area of the factors required for replication and assembly.

Numerous viral pathogens are believed to use or hijack nuclear pore complex (NPC) components to promote their replication, irrespective of a requirement of nucleus functions in their life cycles: particularly, positive-strand RNA viruses such as picornaviruses, rhabdoviruses, retroviruses, and hepaciviruses. Among them, HCV was shown to interfere with localization of some NPC components and to induce their redistribution in the cytoplasm of infected cells, in proximity to LDs, where they partially colocalize with HCV core protein (5, 6). Interestingly, these NPC components seem to play a role in formation of the membranous web by establishing a protective environment for replication (6), which limits the access of pattern recognition receptors (PRRs) to the viral replication and assembly sites (7). Finally, some nucleoporins (Nup98 and Nup54) were identified in a proteomic analysis of purified HCV particles (5), and further investigations confirmed the involvement in Nup98 in HCV assembly (5), even though its precise role remained elusive.

Nup98 is a major constituent of the NPC, a huge protein complex of around 100 to 125 MDa that, by spanning the nuclear envelope, regulates the exchange of proteins and RNA between the nucleus and the cytoplasm. The NPC is composed of multiple copies of about 30 different nucleoproteins (Nups) that are organized in different subcomplexes: the cytoplasmic filament, Y-complex, inner ring complex, transmembrane Nups, central FG Nups, and the nuclear basket (8). The Y-complex, the inner ring, and the transmembrane Nups constitute the scaffold structure, while the FG Nups associate with the NPC-core structure and constitute the permeability barrier. Nup98 is part of the inner ring and is composed of a C-terminal globular region and of an N-terminal disordered region that contains multiple GLFG and FG repeats, as well as binding sites for two mRNA transport factors (RAE1 and TAP/Mex67) (9). The GLFG region participates in the nuclear import of proteins via their interaction with importins and exportins, whereas the mRNA transport binding sites are involved in mRNA export.

Apart from its presence in NPC, Nup98 is also found in nuclear bodies, in which it is supposed to be involved in the regulation of gene expression (9), and in cytoplasmic annulate lamellae (AL) (10). AL are stacked cytoplasmic membranes with inserts of NPC-like structures. They are composed of the full set of NPC scaffold components but lack a subset of other Nups, such as the nuclear basket components and the Nup62 complex (10). The function of AL remains elusive; they could represent cytoplasmic deposits of excess of Nups or, alternatively, preassembled NPCs that could be rapidly mobilized to reform the nuclear envelope during mitosis (11). Interestingly, AL were observed by electron microscopy (EM) at early stages of HCV infection, in close proximity to the precursors of the double-membrane vesicles that allow replication of the HCV viral genome (12), but the precise role of these AL in HCV life cycle is still unknown.

Here, we studied the precise role of Nup98 in HCV assembly by combining biochemical, imaging, and functional analyses. We show that Nup98 is essential for the retention of viral genomic RNAs [(+)RNAs] in subcellular regions promoting replication, assembly, and translation. We also highlight that Nup98 interacts with the viral genome, and we detected a strong apposition of viral genome with Nup98 within infected cells. Finally, we show that the core retrieves Nup98 protein from annulate lamellae, hence allowing an enrichment of Nup98 with viral (+)RNAs in the core region and thus promoting HCV assembly.

## RESULTS

### Nup98 is a proviral factor acting at early HCV assembly steps.

**FIG 1:**
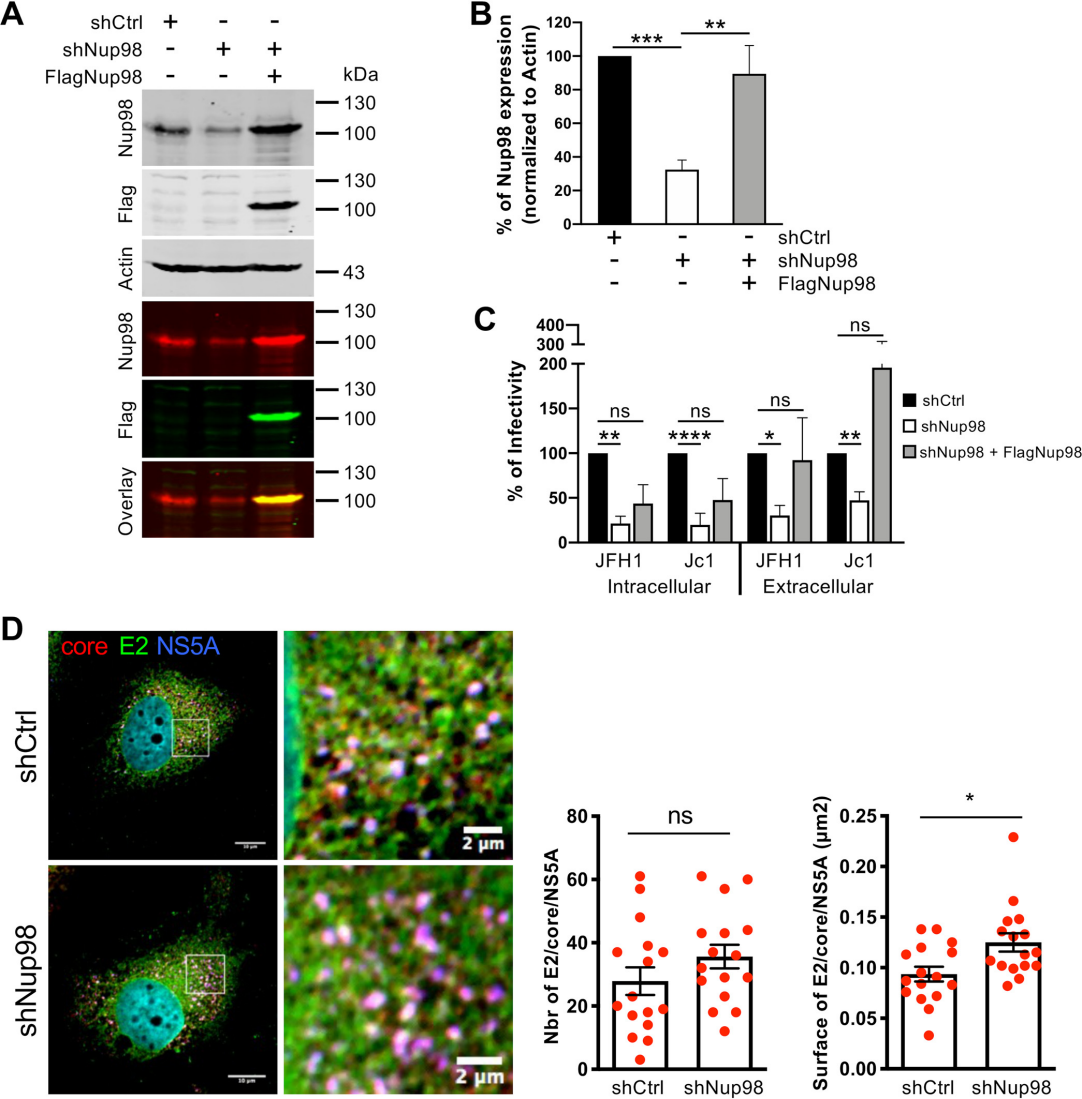
Nup98 is a proviral factor for HCV particle assembly. (A to C) Huh7.5 cells were transduced with lentiviral vectors expressing an empty vector (shCtrl) or an shRNA against Nup98 (shNup98). Transduced cells were then transfected with a plasmid expressing GFP or FlagNup98-sh5res. Eight hours following transfection, cells were infected with Jc1 or JFH1 viruses (MOI of 0.2) and were harvested at 72 h postinfection. (A) Expression of Nup98, Flag, and β-actin proteins was analyzed by Western blotting. The first three Western blot panels are the black and white images of the separated single-channel fluorescent images. The fourth panel is the single-channel fluorescent detection of Nup98 revealed with IRDye 680RD (700-nm red channel). The fifth panel is the single-channel fluorescent detection of Flag revealed with IRDye 800CW (800-nm green channel). The sixth panel is the overlay of the simultaneous detection of Nup98 and Flag. (B) Quantification of Nup98 signals normalized against internal control β-actin signals and expressed as a percentage of Nup98 expression relative to the shCtrl condition. (C) The levels of intracellular and extracellular infectivity were determined from cell lysates or supernatants of infected cells, respectively. (D) Huh7.5 cells transduced with lentiviral vectors expressing an empty vector (shCtrl) or shRNA against Nup98 (shNup98) were infected with Jc1 virus (MOI of 0.2) and were fixed at 72 h postinfection. Following staining for HCV E2, core and NS5A proteins, colocalization of core (red channel) with E2 (green channel) and NS5A (blue channel) proteins was analyzed by confocal microscopy. Colocalized pixels between red, green, and blue channels were extracted with the ColocalizeRGB plugin of ImageJ, and the number and surface of E2/core/NS5A structures were quantified with ImageJ. Scale bars of panels and enlargements from the boxed area represent 10 μm and 2 μm, respectively.

First, we sought to reproduce results from previous studies (5, 6) in order to set up our experimental assays. Hence, we silenced Nup98 expression in Huh7.5 cells by using lentiviral vectors expressing a short hairpin RNA (shRNA) against Nup98 (shNup98), which resulted in up to 10-fold downregulation of Nup98 protein expression (Fig. 1A and B; see Fig. S1A in the supplemental material). We confirmed that Nup98 silencing induces a reduction of both intracellular and extracellular infectious particles, by up to 5-fold for the most potent shRNA, sh5 (Fig. S1B). Of note, since sh2 induced an efficient downregulation of Nup98 (Fig. S1A) but did not significantly impair HCV infectivity (Fig. S1B), we excluded it in our experiments and retained sh5, which was designated shNup98 in all further analyses of this study. We found that shNup98 expression impaired assembly of infectious particles for both JFH1 and Jc1 viruses (Fig. 1C), without affecting HCV replication, as assessed by unchanged levels of intracellular viral RNA (Fig. S1C) and unchanged level of (+)RNA dots in subgenomic replicon (SGR) cells (Fig. S1D).

10.1128/mbio.02923-21.1 FIG S1 Nup98 is a proviral factor for HCV particle assembly. (A and B) Huh7.5 cells transduced with lentiviral vectors expressing different shRNAs against Nup98 or with an empty vector (shCtrl) were infected with Jc1 virus (MOI of 0.2) and were harvested at 72 h postinfection. (A) Expression of Nup98 and β-actin proteins was analyzed by Western blotting. Quantification of Nup98 signals was normalized against internal control β-actin signals and expressed as a percentage of Nup98 expression relative to the shCtrl condition. (B) The levels of intracellular and extracellular infectivity were determined from cell lysates or supernatants of infected cells, respectively. (C) Huh7.5 cells transduced with sh5, the most efficient shRNA against Nup98, or with an empty vector (shCtrl) were infected with Jc1 or JFH1 viruses (MOI of 0.2) and were harvested at 72 h postinfection, and the intracellular genomes were quantified by qPCR. The most efficient shRNA against Nup98 (sh5) is referred to as shNup98 in all subsequent figures. (D) Subgenomic replicon (SGR) cells were transduced with an empty vector (shCtrl) or with sh5 and fixed at 72 h posttransduction. HCV (+)RNA was then stained by fluorescent *in situ* hybridization and analyzed by confocal microscopy. The number of (+)RNA dots per cell was quantified with ImageJ. Download FIG S1, PDF file, 0.1 MB. Copyright © 2022 Boson et al. 2022 Boson et al. https://creativecommons.org/licenses/by/4.0/ This content is distributed under the terms of the Creative Commons Attribution 4.0 International license.

To further confirm the specific effect of Nup98 on HCV assembly, we expressed in Nup98-downregulated cells an shRNA-resistant version of Nup98 fused to an amino-terminal Flag tag (FlagNup98). While this allowed us to restore the level of Nup98 close to that in cells expressing a control shRNA (Fig. 1A and B), we found that it rescued both intracellular infectivity and extracellular infectivity (Fig. 1C), hence confirming the proviral role of Nup98 in HCV assembly.

Next, we sought to understand how Nup98 could promote the early steps of assembly of HCV particles. Specifically, we investigated the formation of E2/core/NS5A clustered structures, which gradually emerge at early time points postinfection in the cytoplasm of infected cells and mark the functional connection of replication complexes to viral particle assembly sites (13). Interestingly, we found that Nup98 downregulation did not prevent the formation of such interfaces but rather induced an increase of their surface, by up to 40% (Fig. 1D). This phenotype is reminiscent of our previous observations that relied on preassembly inhibitors such as daclatasvir, which blocks NS5A-mediated HCV RNA transfer to sites of virion assembly, or diacylglycerol acyltransferase-1 (DGAT-1) inhibitors (13). As shown in this previous study, the increase of the E2/core/NS5A clusters’ size reflects the aggregation of viral proteins that cannot be engaged in efficient viral particle production due to assembly inhibition.

Altogether, these results suggested that Nup98 plays a role in virion production at a step prior to assembly of viral particles.

### Nup98 maintains viral genomic RNA within sites promoting replication, translation, and assembly.

**FIG 2:**
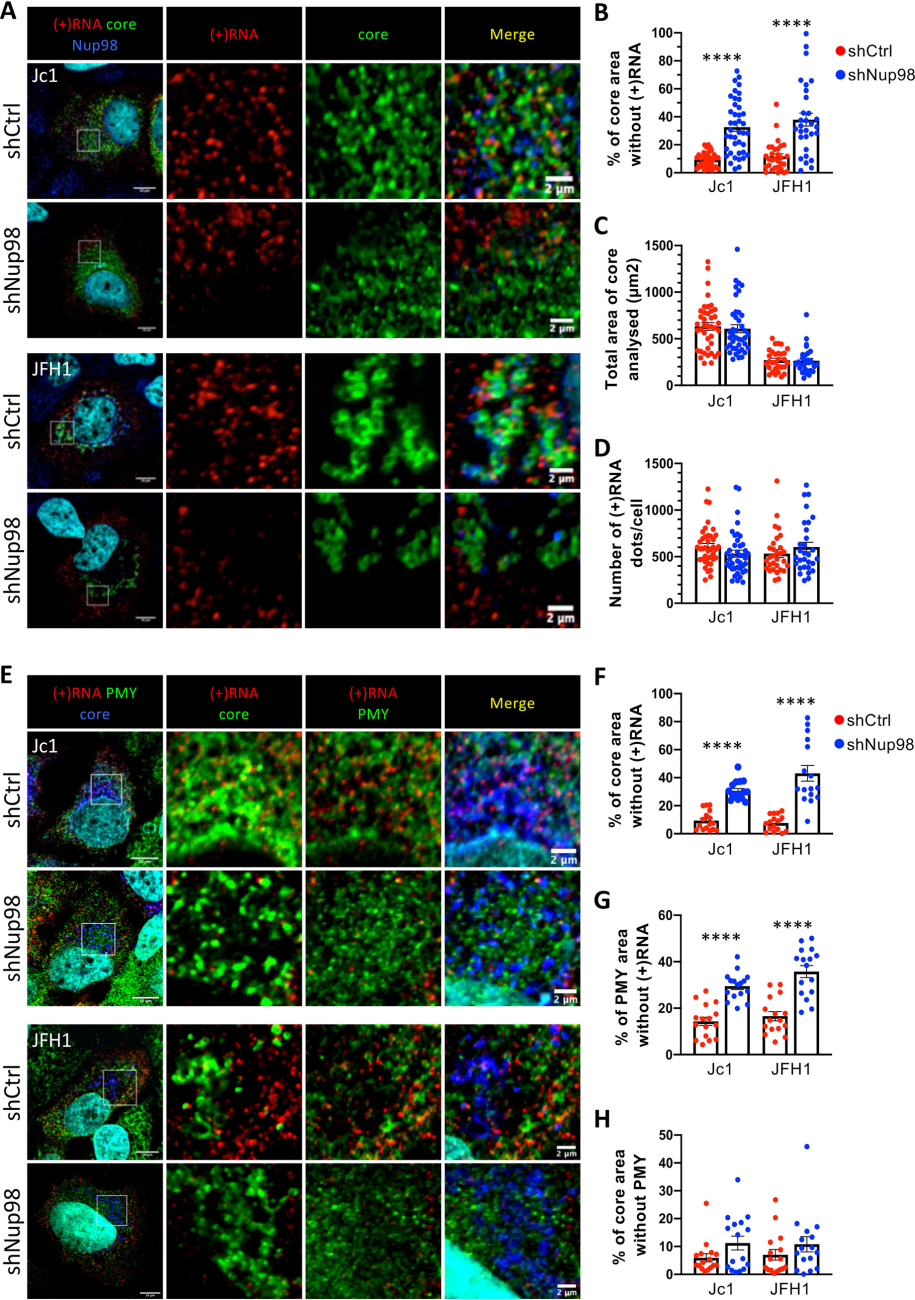
The viral genome is excluded from translation/replication/assembly sites upon Nup98 downregulation. (A to D) Huh7.5 cells transduced with lentiviral vectors expressing an empty vector (shCtrl) or an shRNA against Nup98 (shNup98) were infected with Jc1 or JFH1 viruses (MOI of 0.2) and were fixed at 72 h postinfection. (A) Following staining of Nup98 and HCV core, HCV (+)RNA was stained by fluorescent *in situ* hybridization. Colocalization of Nup98 (blue channel) with core (green channel) and HCV (+)RNA (red channel) was analyzed by confocal microscopy. (B) The percentage of core area without (+)RNA, (C) the total area of core analyzed, and (D) the number of (+)RNA dots per infected cell were quantified with ImageJ. (E to H) Huh7.5 cells expressing shRNA against Nup98 (shNup98) or empty vector (shCtrl) were infected with Jc1 or JFH1 viruses (MOI of 0.2). At 72 h postinfection, active translating ribosomes were labeled with puromycin followed by digitonin extraction, and cells were fixed. Following staining of puromycin and HCV core, HCV (+)RNA was stained by fluorescent *in situ* hybridization. (E) Colocalization of puromycin (PMY) (green channel) with core (blue channel) and HCV (+)RNA (red channel) was analyzed by confocal microscopy. (F) The percentage of core area without (+)RNA, (G) the percentage of puromycin area without (+)RNA, and (H) the percentage of core area without puromycin per infected cell were quantified with ImageJ. Scale bars of panels and enlargements from the boxed area represent 10 μm and 2 μm, respectively.

To address how Nup98 modulates HCV preassembly steps, we investigated the intracellular distribution of HCV genomic and antigenomic RNAs, termed (+)RNAs and (−)RNAs, respectively, and their colocalization with HCV assembly components in Nup98-downregulated cells. Through combined fluorescent *in situ* hybridization (FISH) and immunostaining analyses, we found that HCV (+)RNAs (Fig. 2A to D) and (−)RNAs (see Fig. S2A to D in the supplemental material) were regularly distributed throughout the cytoplasm in JFH1- or Jc1-infected cells. When we quantified the HCV protein-positive areas devoid of viral RNA (Fig. S2E), we found that core and NS5A unfrequently exhibited intracellular regions that lacked HCV (+)RNA at 72 h postinfection (Fig. 2A to D; see Fig. S3A to D in the supplemental material), thus showing their abundant colocalization. Strikingly, we found that Nup98 downregulation induced a partition of the viral (+)RNA. Indeed, although the density and perinuclear localization of HCV core or NS5A were not changed upon Nup98 silencing, we found that HCV (+)RNA localization was mostly restricted to distal cellular areas, where core and NS5A are generally not densely located (Fig. 2A to D; see Fig. S3A to D). More specifically, the core or NS5A areas occupied the same intracellular surfaces, and we detected the same number of (+)RNAs per infected cell for either condition; yet, ca. 40% of core region was devoid of (+)RNA upon downregulation of Nup98 (versus 10% in control Huh7.5 cells) (Fig. 2A to D; Fig. S3A to D).

10.1128/mbio.02923-21.2 FIG S2 The viral genome is excluded from translation/replication/assembly sites upon Nup98 downregulation. (A to D) Huh7.5 cells transduced with lentiviral vectors expressing an empty vector (shCtrl) or an shRNA against Nup98 (shNup98) were infected with Jc1 or JFH1 viruses (MOI of 0.2) and were fixed at 72 h postinfection. (A) Following staining of Nup98 and HCV core, HCV (−)RNAs were stained by fluorescent *in situ* hybridization. Colocalization of Nup98 (green channel) with core (red channel) and HCV (−)RNA (blue channel) was analyzed by confocal microscopy. (B) The percentage of core area without (−)RNA, (C) the total area of core analyzed, and (D) the number of RNA dots per infected cell were quantified with ImageJ. Scale bars of panels and enlargements from the boxed area represent 10 μm and 2 μm, respectively. (E) Pipeline of the segmentation analysis used in Fig. 2 and Fig. S1 and 2. The confocal images were first split with ImageJ software, and a Gaussian blur with a sigma of 10 was applied on the cells of interest in each channel. An autothresholding with the Dark Huang method was then applied, followed by a conversion into masks. The masks encompass the regions occupied by each cellular or viral marker, and different parameters were recorded inside or outside the masks with the “Analyze Particles” function: area of each mask, number of objects, and common area between 2 masks. Download FIG S2, PDF file, 0.4 MB. Copyright © 2022 Boson et al. 2022 Boson et al. https://creativecommons.org/licenses/by/4.0/ This content is distributed under the terms of the Creative Commons Attribution 4.0 International license.

10.1128/mbio.02923-21.3 FIG S3 Nup98 maintains the viral genome near replication/assembly sites. Huh7.5 cells transduced with lentiviral vectors expressing an empty vector (shCtrl) or an shRNA against Nup98 (shNup98) were infected with Jc1 or JFH1 viruses (MOI of 0.2) and were fixed at 72 h postinfection. Following staining of HCV core and NS5A, HCV (+)RNA (A to D) and (−)RNA (E to H) species were stained by fluorescent *in situ* hybridization. Colocalization of NS5A (blue channel) with core (green channel) and HCV (+)RNA (red channel) (A) or NS5A (green channel) with core (red channel) and HCV (−)RNA (blue channel) (E) was analyzed by confocal microscopy. For clarity, the enlarged areas are pseudocolored red for (+)RNA and (−)RNA and green for NS5A. The percentage of NS5A area without RNA species (B and F), the total area of NS5A analyzed (C and G), and the number of RNA dot species (D and H) per infected cell were quantified with ImageJ. Scale bars of panels and enlargements from the boxed area represent 10 μm and 2 μm, respectively. Download FIG S3, PDF file, 0.5 MB. Copyright © 2022 Boson et al. 2022 Boson et al. https://creativecommons.org/licenses/by/4.0/ This content is distributed under the terms of the Creative Commons Attribution 4.0 International license.

We then sought to address the specificity of this (+)RNA partitioning. First, by assessing HCV (−)RNA that were unfrequently associated with core or NS5A, we did not observe an alteration of their localization upon Nup98 downregulation (Fig. S2A to D; Fig. S3E to H). Second, when we investigated an irrelevant RNA control consisting in an ectopic mRNA encoding a nanoLuc luciferase sequence via a lentiviral vector (nLuc) that was expressed at levels similar to those of HCV (+)RNAs (compare RNA dots per cell in Fig. 2D with those in Fig. S4D in the supplemental material), we found that alike for HCV (−)RNA, ca. 40% of the core region did not colocalize with this nLuc RNA (Fig. S4A to C), irrespective of the presence of Nup98. This suggested first that when Nup98 is downregulated, HCV (+)RNA behaves like alternative RNAs such as, e.g., mRNAs, and second that Nup98 is required to specifically maintain HCV (+)RNA in core/NS5A-containing areas.

10.1128/mbio.02923-21.4 FIG S4 The RNA partitioning upon Nup98 downregulation is specific to HCV (+)RNA and is a progressive event. Huh7.5 cells transduced with lentiviral vectors expressing an empty vector (shCtrl) or an shRNA against Nup98 (shNup98) were transduced with a lentiviral vector expressing the nLuc reporter gene, infected or not with Jc1 or JFH1 viruses, and fixed at 72 h postinfection. Following immunostaining of core, the nLuc RNA and HCV (+)RNA were stained by fluorescent *in situ* hybridization. (A) Colocalization of core (blue channel) with the nLuc RNA (green channel) and HCV (+)RNA (red channel) was analyzed by confocal microscopy. (B) The percentage of core area without nLuc RNA and (C) the number of nLuc RNA dots per infected cell were quantified with ImageJ. Scale bars of panels and enlargements from the boxed area represent 10 μm and 2 μm, respectively. (D and E) Huh7.5 cells transduced with lentiviral vectors expressing an empty vector (shCtrl) or an shRNA against Nup98 (shNup98) were infected with Jc1 virus (MOI of 0.2) and were fixed at 24, 48, or 72 h postinfection. Following staining of HCV core and NS5A, HCV (+)RNA was stained by fluorescent *in situ* hybridization, and the colocalization of (+)RNA with core or NS5A was analyzed by confocal microscopy. (D) The percentage of core area without (+)RNA or (E) the percentage of NS5A area without (+)RNA was quantified with ImageJ. Download FIG S4, PDF file, 1.7 MB. Copyright © 2022 Boson et al. 2022 Boson et al. https://creativecommons.org/licenses/by/4.0/ This content is distributed under the terms of the Creative Commons Attribution 4.0 International license.

Next, we addressed the kinetics of the HCV (+)RNA aggregation and segregation in and from core/NS5A areas, respectively. We found that the proportion of core-positive areas devoid of (+)RNA progressively decreased over time (i.e., by 3-fold from 24 h to 48 h postinfection), resulting in ca. 5% of core regions lacking (+)RNA at late time points postinfection (Fig. S4D). In contrast, in Nup98-downregulated cells, we found that the core areas were progressively cleared from HCV (+)RNAs, resulting in over 45% of the core regions devoid of (+)RNAs at 72 h postinfection (Fig. S4D). Similar observations were obtained with NS5A (Fig. S4E). Altogether, these results suggested that HCV hijacks Nup98 early and progressively to help maintain (+)RNAs near replication/assembly sites.

Finally, to better define the areas in which viral proteins versus (+)RNAs are partitioned in HCV-infected cells upon Nup98 silencing, we analyzed their intracellular localization with respect to active translation sites, using a subcellular ribopuromycylation assay (14). This assay is based on immunofluorescent (IF) detection of puromycin (PMY), an aminoglycoside antibiotic that mimics charged tRNA^Tyr^, which is incorporated into nascent polypeptide chains upon its entry in the ribosome A site. Intracellular PMY staining of HCV-infected cells revealed cells exhibiting either a high translational activity and no infection or, alternatively, poor translational activity and expression of HCV infection markers, as judged by expression only in the latter cells of HCV core protein and (+)RNAs (see Fig. S5 in the supplemental material). Despite this overall lower translation activity typical of HCV-infected cells (15), which appeared independent of Nup98 expression levels (Fig. S5), active ribosomes were found spread throughout the cell, in agreement with the broad distribution of HCV (+)RNA (Fig. 2E). Interestingly, we found that upon downregulation of Nup98 that excluded the HCV (+)RNA from core-positive regions (Fig. 2A to D), PMY-positive areas remained detectable within the perinuclear region occupied by core and, accordingly, were devoid of (+)RNA (Fig. 2E to H). This suggested that, in the absence of Nup98, the area occupied by core and NS5A proteins maintains an active translational activity.

10.1128/mbio.02923-21.5 FIG S5 HCV infection decreases the overall cellular translation. Huh7.5 cells transduced with lentiviral vectors expressing an empty vector (shCtrl) or an shRNA against Nup98 (shNup98) were infected with Jc1 or JFH1 viruses (MOI of 0.2). At 72 h postinfection, active translating ribosomes were labeled with puromycin followed by digitonin extraction, and cells were fixed. Following staining of puromycin and HCV core, HCV (+)RNAs were stained by fluorescent *in situ* hybridization. Colocalization of puromycin (PMY) (green channel) with core (blue channel) and HCV (+)RNA (red channel) was analyzed by confocal microscopy. Individual channels were pseudocolored white, and infected cells were labeled with yellow stars. Scale bars of merged panels represent 30 μm. Download FIG S5, PDF file, 2.3 MB. Copyright © 2022 Boson et al. 2022 Boson et al. https://creativecommons.org/licenses/by/4.0/ This content is distributed under the terms of the Creative Commons Attribution 4.0 International license.

Altogether, our results suggested that Nup98 helps to maintain HCV (+)RNA at intracellular regions of translation, replication, and assembly. Thus, by concentrating these viral RNAs within these areas, Nup98 may favor the generation of new replication organelles and the assembly of new progeny virions.

### Nup98 interacts with HCV RNA and is frequently apposed to HCV (+)RNA dot structures.

**FIG 3:**
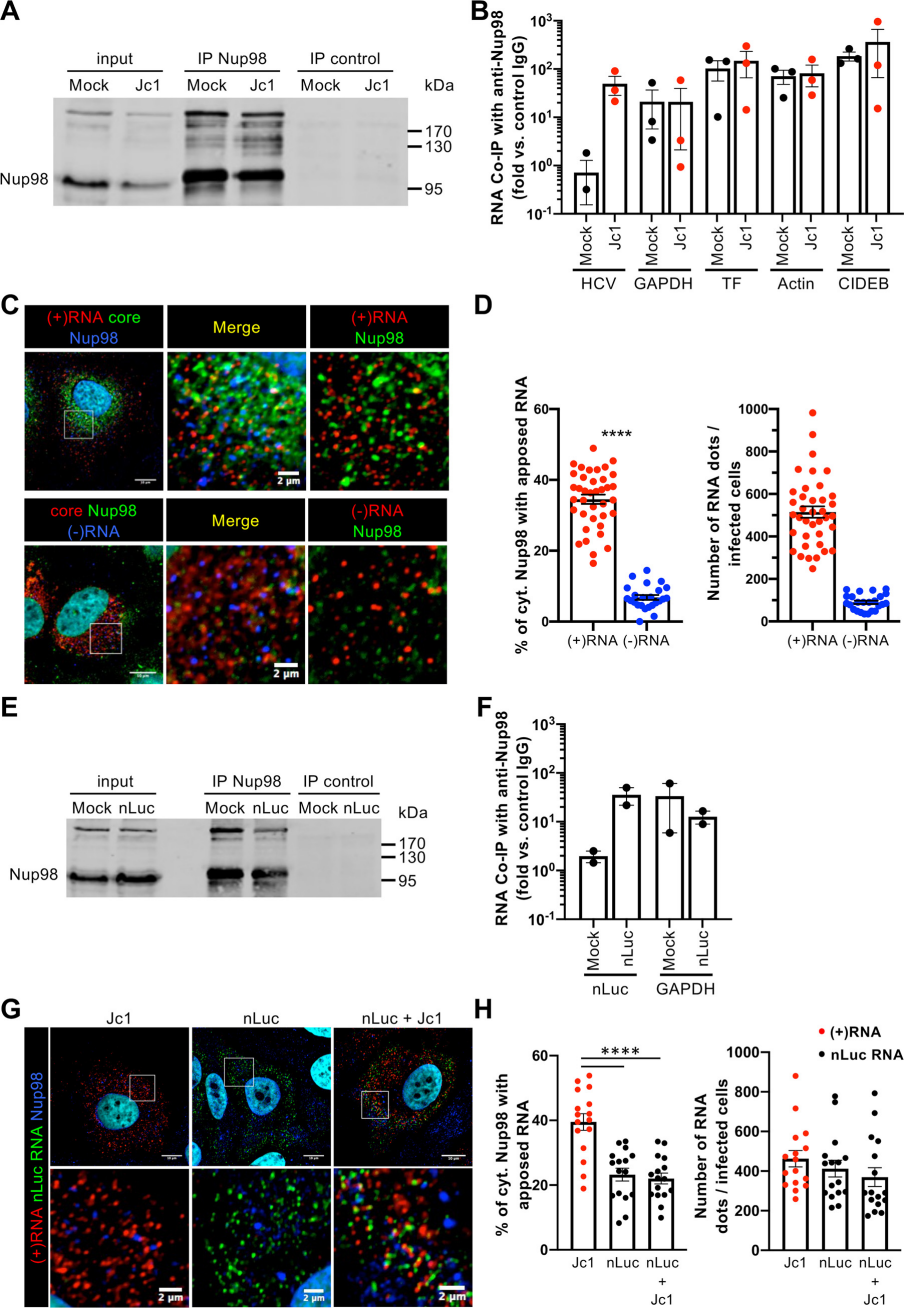
Nup98 is apposed to (+)RNA. (A and B) Huh7.5 cells infected with Jc1 (MOI of 0.5) were subjected to immunoprecipitation against endogenous Nup98 with anti-Nup98 antibody (IP Nup98) or control immunoglobulins (IP control). (A) The efficiency of immunoprecipitation was analyzed by Western blotting. (B) HCV RNA and cellular GAPDH, transferrin (TF), actin, and CIDEB RNAs coimmunoprecipitated with Nup98 were quantified by qPCR. Of note, we observed a slight difference in molecular weight of Nup98 in immunoprecipitation lanes, which was probably due to a specific capture of posttranslationally modified and cytoplasmic Nup98 (55). The specific capture of RNA is expressed as the fold enrichment of RNA bound to Nup98 immunoprecipitated with the Nup98 antibody against control immunoglobulins (rat IgG). (C and D) Huh7.5 cells infected with Jc1 virus (MOI of 0.2) were fixed at 72 h postinfection. Following immunostaining of Nup98 and HCV core, HCV (+)RNA and (−)RNA species were stained by fluorescent *in situ* hybridization. (C) Colocalization of Nup98 (blue channel) with core (green channel) and HCV (+)RNA (red channel) (top row) or Nup98 (green channel) with core (red channel) and HCV (−)RNA (blue channel) (bottom row) was analyzed by confocal microscopy. For clarity, the enlarged areas showing apposition (right panels) are pseudocolored red for (+)RNA and (−)RNA and green for Nup98 (green). (D) The percentage of cytoplasmic Nup98 with apposed RNA and the number of RNA dots of each RNA species per infected cells were quantified with ImageJ. (E and F) Huh7.5 cells transduced with nLuc lentiviral vector (MOI of 10) were subjected to immunoprecipitation against endogenous Nup98 with anti-Nup98 antibody (IP Nup98) or control immunoglobulins (IP control). (E) The efficiency of immunoprecipitation was analyzed by Western blotting. (F) nLuc RNA (nLuc) and cellular GAPDH RNAs coimmunoprecipitated with Nup98 were quantified by qPCR. (G to I) Huh7.5 cells were transduced with nLuc lentiviral vector, infected or not with Jc1 virus, and fixed at 72 h postinfection. Following immunostaining of Nup98, the nLuc mRNA and HCV (+)RNA were stained by fluorescent *in situ* hybridization. (G) Colocalization of Nup98 (blue channel) with the nLuc RNA (green channel) and HCV (+)RNA (red channel) was analyzed by confocal microscopy. (H) The percentage of cytoplasmic Nup98 with apposed HCV (+)RNA or nLuc RNA and the number of HCV (+)RNA or nLuc RNA dots per cell were quantified with ImageJ. Scale bars of panels and enlargements from the boxed area represent 10 μm and 2 μm, respectively.

Since Nup98 harbors an RNA-binding domain (16), we wondered if Nup98 could interact with viral RNAs in HCV-infected cells. First, we sought to address whether Nup98 directly interacts with HCV RNA. Hence, we immunoprecipitated Nup98 in Jc1-infected cells (Fig. 3A) and quantified by reverse transcription-quantitative PCR (RT-qPCR) coimmunoprecipitated HCV RNA or mRNAs from an array of cellular genes, such as those coding for GAPDH (glyceraldehyde-3-phosphate dehydrogenase), transferrin (TF), actin, and CIDEB. Likely owing to the presence of its RNA-binding domain, we found that Nup98 could readily coimmunoprecipitate these cellular mRNAs, in both infected and noninfected cells (Fig. 3B). Importantly, we found that Nup98 could also efficiently capture HCV RNA, at levels similar to those of cellular RNAs (Fig. 3B). This indicated that Nup98 may interact with the HCV RNA genome.

Next, we wondered if Nup98 could be detected in proximity to viral RNA at the single-cell level. We distinguished Nup98 located at the nuclear pore versus in the cytosol, which appeared as dots throughout the cytoplasm in both Jc1 and JFH1 cell culture-grown HCV (HCVcc)-infected cells (Fig. 3C; see Fig. S6A in the supplemental material). Consistently, we detected perinuclear regions in which Nup98 and HCV (+)RNA were abundantly colocalized (Fig. 3C and D; Fig. S6A and B). Specifically, we found that ca. 40% of Nup98 dots exhibited apposed dots of HCV (+)RNA, suggesting their close proximity. In contrast, we found that HCV (−)RNA dots were not frequently apposed to Nup98 dots (Fig. 3C and D; Fig. S6A and B). This result led us to address the specificity of the apposition to Nup98 detected with HCV (+)RNA.

10.1128/mbio.02923-21.6 FIG S6 Nup98 is apposed to (+)RNA. (A and B) Huh7.5 cells infected with JFH1 virus (MOI of 0.2) were fixed at 72 h postinfection. Following immunostaining of Nup98 and HCV core, HCV (+)RNA and (−)RNA species were stained by fluorescent *in situ* hybridization. (A) Colocalization of Nup98 (blue channel) with core (green channel) and HCV (+)RNA (red channel) (top row) or Nup98 (green channel) with core (red channel) and HCV (−)RNA (blue channel) (bottom row) was analyzed by confocal microscopy. For clarity, the enlarged areas showing apposition (right panels) are pseudocolored red for (+)RNA and (−)RNA and green for Nup98. (B) The percentage of cytoplasmic Nup98 with apposed RNA and the number of RNA dots of each RNA species per infected cells were quantified with ImageJ. (C and D) Huh7.5 cells infected with Jc1 virus (MOI of 0.2) or mock infected were fixed at 72 h postinfection. Following immunostaining of Nup98, HCV (+)RNA and actin or GAPDH RNA was stained by fluorescent *in situ* hybridization. (C) Colocalization of Nup98 (blue channel) with HCV (+)RNA (red channel) and actin or GAPDH RNA (green channel) was analyzed by confocal microscopy. (D) The percentage of cytoplasmic Nup98 with apposed RNA and the number of RNA dots of each RNA species per cell were quantified with ImageJ. (E and F) Huh7.5 cells were transduced with nLuc vector, infected, or not, with JFH1 virus, and fixed at 72 h postinfection. Following immunostaining of Nup98, the nLuc RNA and HCV (+)RNA were stained by fluorescent in situ hybridization. (E) Colocalization of Nup98 (blue channel) with the nLuc RNA (green channel) and HCV (+)RNA (red channel) was analyzed by confocal microscopy. (F) The percentage of cytoplasmic Nup98 with apposed RNA and the number of RNA dots of each RNA species per cell were quantified with ImageJ. Scale bars of panels and enlargements from the boxed area represent 10 μm and 2 μm, respectively. Download FIG S6, PDF file, 2.6 MB. Copyright © 2022 Boson et al. 2022 Boson et al. https://creativecommons.org/licenses/by/4.0/ This content is distributed under the terms of the Creative Commons Attribution 4.0 International license.

While Nup98 interacts with actin and GAPDH RNAs (Fig. 3B), we found that only ca. 10% of Nup98 was apposed to these latter RNAs, in contrast to the 5- to 6-fold-higher Nup98 apposition detected with HCV (+)RNA (Fig. S6C and D). Due to the large excess of HCV RNA levels compared to actin or GAPDH RNA (Fig. S6D), which may bias the latter results, we thought to determine the apposition of Nup98 with nLuc RNA, which is expressed at similar levels to HCV (+)RNA (Fig. 3H) and which interacts with Nup98 (Fig. 3E and F). Yet, while these latter RNAs were regularly distributed throughout the cytoplasm (Fig. 3G; Fig. S6E), we found that they were less abundantly apposed to cytoplasmic Nup98, compared to HCV (+)RNA apposition to Nup98 [23% versus 40% for Jc1 (+)RNA or 47% for JFH1 (+)RNA] (compare Fig. 3H with Fig. 3D or Fig. S6B) and that Jc1 or JFH1 HCVcc infection did not alter their Nup98 apposition level (Fig. 3G and H; Fig. S6E and F).

Altogether, these results suggested that Nup98 can interact with HCV (+)RNA, as well as with non-HCV RNAs, but can be readily detected in close proximity to HCV (+)RNA, hence suggesting a viral mechanism allowing Nup98 and HCV (+)RNA apposition.

### Cytosolic Nup98 localization and its apposition to viral RNA are regulated by core protein.

Since Nup98 interacts with HCV (+)RNAs (Fig. 3) and is required to allow their retention in core- and S5A-positive areas (Fig. 2A to D; Fig. S3A to D), we hypothesized that core, which can interact with Nup98 (5), could influence Nup98 localization to induce HCV RNA retention in sites promoting replication, translation, and assembly.

**FIG 4:**
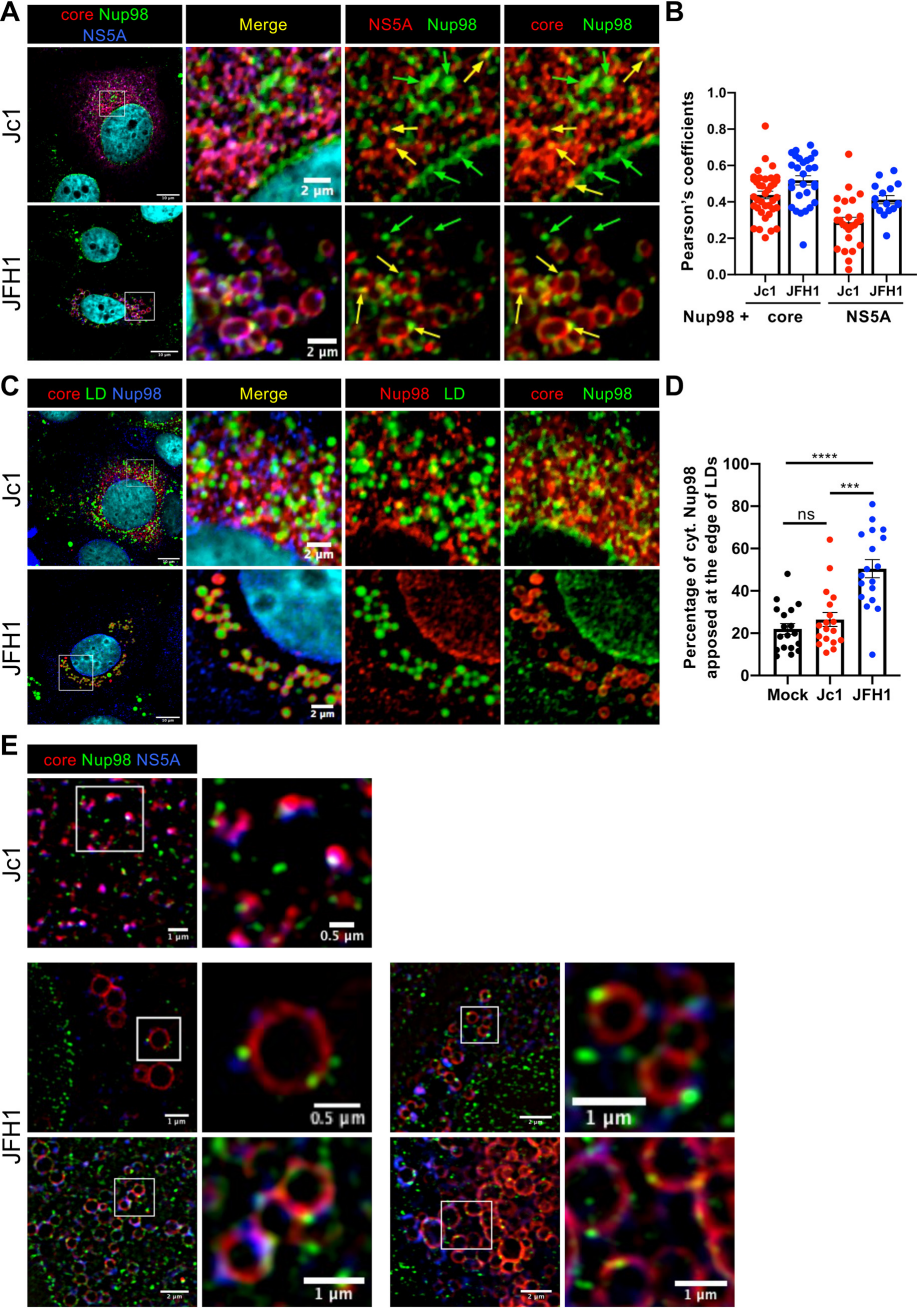
Nup98 colocalizes with HCV proteins. Huh7.5 cells infected with Jc1 or JFH1 viruses (MOI of 0.2) were fixed at 72 h postinfection. (A) Nup98 and HCV core and NS5A proteins were immunostained, and the colocalization of Nup98 (green channel) with core (red channel) and NS5A (blue channel) was analyzed by confocal microscopy. For clarity, the enlarged areas showing double colocalization (right panels) are pseudocolored red for NS5A or core and green for Nup98, and the colocalization spots are shown with yellow arrows. (B) The degree of colocalization between cytoplasmic Nup98 and core or NS5A was assessed by determining the Pearson’s correlation coefficients with the JACoP plugin of ImageJ. (C) Nup98, lipid droplets, and HCV core protein were immunostained, and the colocalization of lipid droplets (LD) (green channel) with core (red channel) and Nup98 (blue channel) was analyzed by confocal microscopy. For clarity, the enlarged areas showing double colocalization (right panels) are pseudocolored red for Nup98 and green for LD or red for core and green for Nup98. (D) The percentage of cytoplasmic Nup98 apposed at the edge of LDs was quantified with ImageJ. Scale bars of panels and enlargements from the boxed area represent 10 μm and 2 μm, respectively. (E) Nup98 and HCV core and NS5A proteins were immunostained, and the colocalization of Nup98 (green channel) with core (red channel) and NS5A (blue channel) was analyzed by 3D-SIM superresolution microscopy.

First, to address this possibility, we investigated the intracellular sites of colocalization of Nup98 with core and NS5A (Fig. 4). Cytoplasmic Nup98 was detected in the cytoplasm of Jc1 and JFH1 HCVcc-infected cells, where it colocalized with HCV core and NS5A (yellow arrows in Fig. 4A), as judged by the Pearson’s correlation coefficients (Fig. 4B), which agreed with results of others (5, 6). Moreover, we observed in JFH1 virus-infected cells a particularly well-defined distribution pattern for Nup98 and either viral protein around or apposed to round structures of ca. 1 to 2 μm (Fig. 4A; see Fig. S7A in the supplemental material) that appeared to be lipid droplets (LDs). Of note, previous studies showed that the core protein detected in JFH1 HCVcc-infected cells accumulates at the surface of LDs, whereas Jc1 core is frequently apposed to LDs without covering these organelles in infected cells (4, 13, 17, 18). Yet, when we determined the localization of Nup98 with respect to LDs (Fig. 4C and D; Fig. S7B), we found that while Nup98 was not readily localized around LDs in mock-infected cells or in Jc1 HCVcc-infected cells, Nup98 was specifically found apposed around LDs in JFH1 HCVcc-infected cells. Using the superresolution microscopy technique 3D-SIM (three-dimensional structured illumination microscopy), we confirmed that Nup98 was apposed as discrete dots at the edge of LDs that were covered by core protein in JFH1 virus-infected cells (Fig. 4E). Hence, these results indicated that HCV infection could alter Nup98 intracellular localization in a strain-specific manner.

10.1128/mbio.02923-21.7 FIG S7 Nup98 colocalizes with HCV proteins. Huh7.5 cells infected with Jc1 or JFH1 viruses (MOI of 0.2) were fixed at 72 h postinfection. (A) Nup98 and HCV core and NS5A proteins were immunostained, and the colocalization of Nup98 (green channel) with core (red channel) and NS5A (blue channel) was analyzed by confocal microscopy. For clarity, the enlarged areas showing single channels were pseudocolored in grayscale. (B) Nup98, lipid droplets and HCV core protein were immunostained, and the colocalization of lipid droplets (LD) (green channel) with core (red channel) and Nup98 (blue channel) was analyzed by confocal microscopy. For clarity, the enlarged areas showing single channels were pseudocolored in grayscale. Scale bars of panels and enlargements from the boxed area represent 10 μm and 2 μm, respectively. Download FIG S7, PDF file, 2.8 MB. Copyright © 2022 Boson et al. 2022 Boson et al. https://creativecommons.org/licenses/by/4.0/ This content is distributed under the terms of the Creative Commons Attribution 4.0 International license.

**FIG 5:**
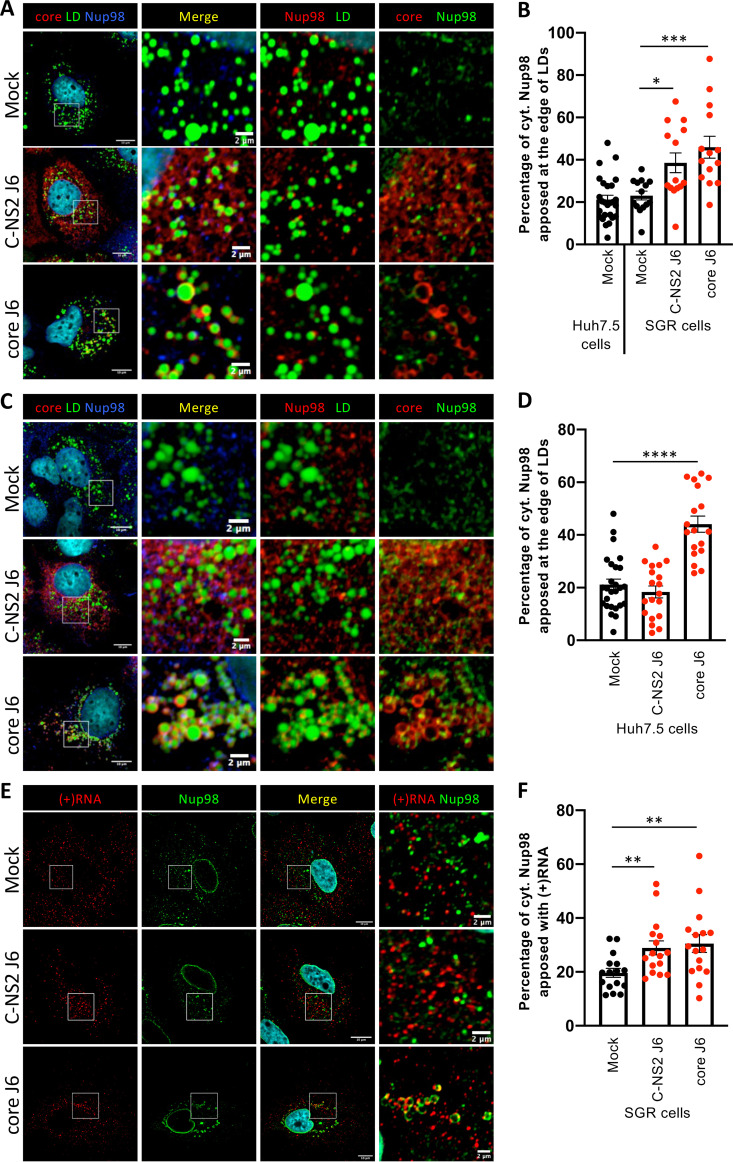
HCV core redirects Nup98 near LDs independently of other viral factors. Subgenomic replicon cells (SGR cells) (A, B, E, and F) or Huh7.5 cells (C and D) were transduced with lentiviral vectors expressing core or core to NS2 (C-NS2) from the J6 HCV strain and were fixed at 72 h postransduction. (A and C) Lipid droplets (LD), Nup98, and HCV core were stained, and the colocalization of Nup98 (blue channel) with core (red channel) and LD (green channel) was analyzed by confocal microscopy. For clarity, the enlarged areas showing apposition (right panels) are pseudocolored red for Nup98 and green for LD or red for core and green for Nup98. (B and D) The percentage of cytoplasmic Nup98 apposed at the edge of LDs was quantified with ImageJ. (E) Following immunostaining of Nup98 (green channel), HCV (+)RNA was stained by fluorescent *in situ* hybridization (red channel), and apposition was analyzed by confocal microscopy. (F) The percentage of cytoplasmic Nup98 apposed with (+)RNA was quantified with ImageJ. Scale bars of panels and enlargements from the boxed area represent 10 μm and 2 μm, respectively.

Next, since the core protein does not accumulate around LDs in Jc1 HCVcc-infected cells, in contrast to JFH1 HCVcc-infected cells (4, 13, 17, 18), the above results suggested that core influences Nup98 localization. To confirm this possibility, we transduced SGR-expressing Huh7.5 cells (19) with lentiviral vectors expressing either core alone, which is thus targeted around LDs, or a core-NS2 polyprotein, which modulates core localization at LDs in an HCV strain-dependent manner (17). Compared to naive Huh7.5 cells, SGR expression did not change Nup98 localization, which appeared both at the nuclear membrane and within the cytoplasm (Fig. 5A and B). Yet, we found that individual core expression or JFH1 core-NS2 expression in SGR cells induced the relocalization of Nup98 in discrete punctate structures apposed to LDs (data not shown), similar to those detected in JFH1 HCVcc-infected cells, whereas expression of J6 core-NS2 polyprotein, which does not induce core localization at LDs (17), only partially relocalized Nup98 at LDs (Fig. 5A and B). Importantly, similar results were obtained when core versus core-NS2 proteins were expressed in naive Huh7.5 cells (i.e., in the absence of SGR-mediated expression of NS3-NS5B proteins and HCV RNAs) (Fig. 5C and D), thus confirming that core expression alone is sufficient to redistribute Nup98 within cells and that neither alternative viral factors nor virus-induced cellular modifications (e.g., membrane remodeling induced by infection or SGR expression) (20, 21) influence core-mediated Nup98 relocalization.

Then, we investigated the role of core on apposition of Nup98 with HCV (+)RNA. Whereas in SGR cells apposition of Nup98 with viral (+)RNAs was comparable to that of non-viral RNA (Fig. 5E and F, compare “Mock” with Fig. 3H), we found that the presence of core was necessary and sufficient to increase the apposition of Nup98 with (+)RNA (Fig. 5E and F) to a level close to that of HCV-infected cells (Fig. 3C and D), confirming that core alone can induce Nup98 relocalization close to viral components. Of note, we confirmed the interaction of Nup98 with HCV or with cellular mRNAs in the context of SGR cells (see Fig. S8A and B in the supplemental material), confirming that Nup98 could associate at a basal level with viral RNA as well as with cellular RNAs and indicating that core can favor the apposition of viral (+)RNAs with Nup98. Finally, we also showed that core is necessary and sufficient to induce retention of HCV (+)RNAs in an NS5A-positive region (Fig. S8C).

10.1128/mbio.02923-21.8 FIG S8 Nup98 coprecipitates HCV RNA in SGR cells. (A and B) Subgenomic replicon (SGR) cells transduced with core to NS2 (C-NS2) lentiviral vectors from the J6 strain were subjected to immunoprecipitation against endogenous Nup98 with anti-Nup98 antibody or control immunoglobulins (rat IgG). (A) The efficiency of the immunoprecipitation was analyzed by Western blotting. (B) HCV RNA and cellular GAPDH RNA coimmunoprecipitated with Nup98 were quantified by qPCR. The specific capture of RNA is expressed as the fold enrichment of RNA bound to Nup98 immunoprecipitated with the Nup98 antibody against control immunoglobulins (rat IgG). (C) SGR cells expressing shRNA against Nup98 (shNup98) or empty vector (shCtrl) were transduced with lentiviral vectors expressing core or C-NS2 from the J6 HCV strain and were fixed at 72 h posttransduction. Following staining of HCV core and NS5A, HCV (+)RNA was stained by fluorescent *in situ* hybridization. Colocalization of NS5A (blue channel) with core (green channel) and HCV (+)RNA (red channel) was analyzed by confocal microscopy. Scale bars of panels and enlargements from the square area represent 10 μm and 2 μm, respectively. Download FIG S8, PDF file, 2.5 MB. Copyright © 2022 Boson et al. 2022 Boson et al. https://creativecommons.org/licenses/by/4.0/ This content is distributed under the terms of the Creative Commons Attribution 4.0 International license.

Altogether, these results indicated that individual core expression can regulate Nup98 localization in the cytosol and that core protein colocalizes with Nup98, hence suggesting that core may recruit Nup98 and induce its proximity with HCV (+)RNAs.

### Nup98 is retrieved by the HCV core protein from annulate lamellae, independently of karyopherins.

We then sought to determine if HCV core retrieves Nup98 alone or in combination with other proteins from the nuclear pore complex (NPC) or, alternatively, from annulate lamella (AL) pore complex (ALPC). The latter structures are discrete cytosolic double-membrane structures containing most components of the NPC, with the exception of some Nups such as TPR (also known as Megator/Mtor), a nuclear basket protein (10), and were identified in close proximity to HCV replication organelles (12). While these structures have mainly been identified by electron microscopy (22), recent studies indicated that RanBP2 (also known as Nup358) is a major constituent of ALPC (10, 23), together with Nup98 (10), and that cytoplasmic RanBP2 staining could be considered a reliable AL marker (24). We therefore used TPR as a marker of NPC and RanBP2 as a marker of ALPC in subsequent immunofluorescence assays.

**FIG 6:**
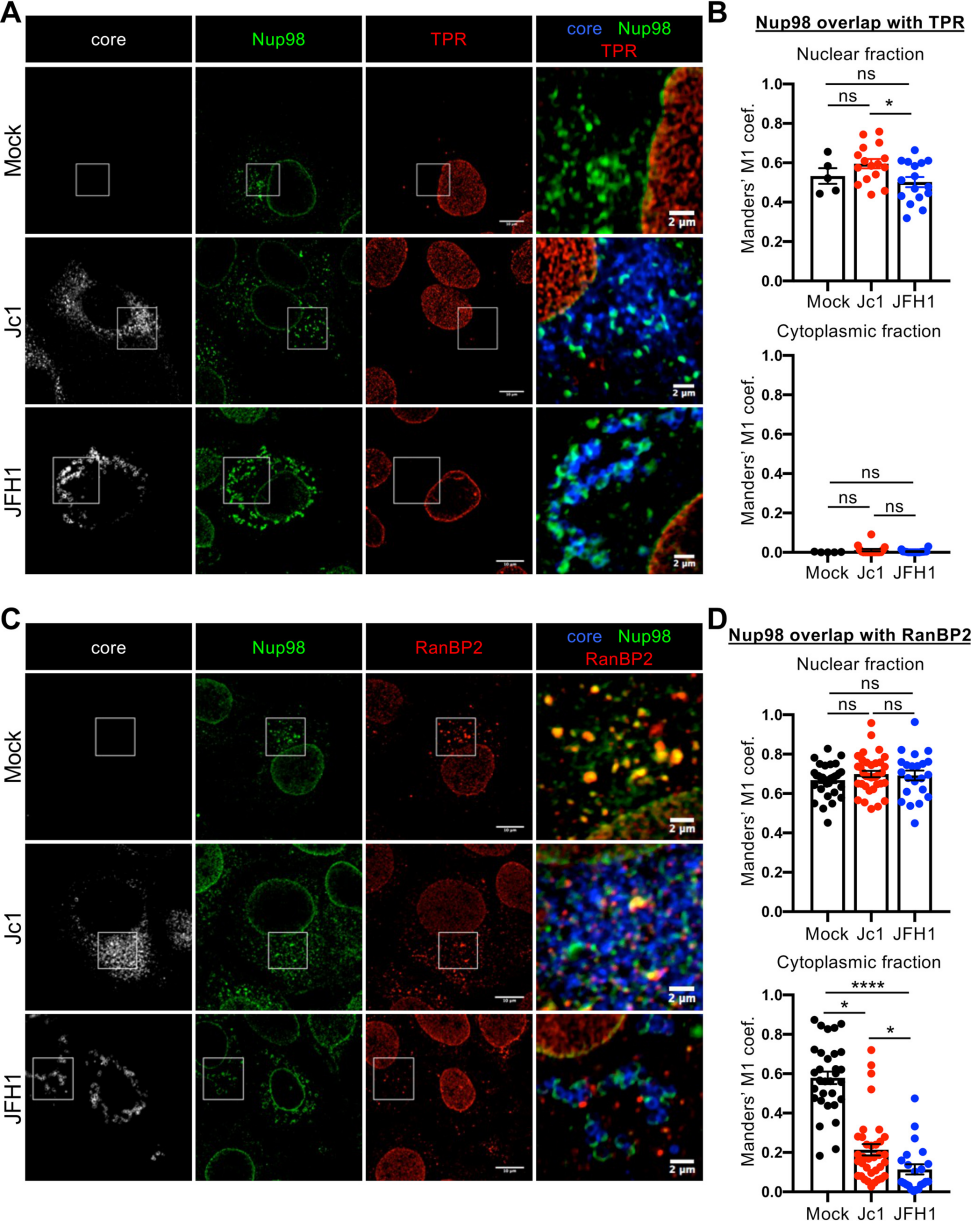
Nup98 is retrieved from annulate lamellae by core protein. Huh7.5 cells infected with Jc1 or JFH1 viruses (MOI of 0.2) were fixed at 72 h postinfection. (A and C) Nup98, HCV core, and TPR or RanBP2 proteins were immunostained, and the colocalization of Nup98 (green channel) with core (red channel) and TPR (A) or RanBP2 (C) (blue channels) was analyzed by confocal microscopy. (B and D) The degree of overlap between nuclear or cytoplasmic Nup98 and TPR or RanBP2 was assessed by quantifying the Manders’ M1 overlap coefficients, representing the overlap of Nup98 signal with TPR (B) or RanBP2 (D) signals in the different fractions, with the JACoP plugin of ImageJ. Scale bars of panels and enlargements from the boxed area represent 10 μm and 2 μm, respectively.

First, we found that TPR, which readily colocalized with Nup98 at the nuclear membrane (Fig. 6A), was not relocalized to core/Nup98 cytoplasmic areas in HCVcc-infected cells (Fig. 6A and B), thus ruling out that Nup98 relocalization within core/NS5A area would form a bona fide NPC.

**FIG 7:**
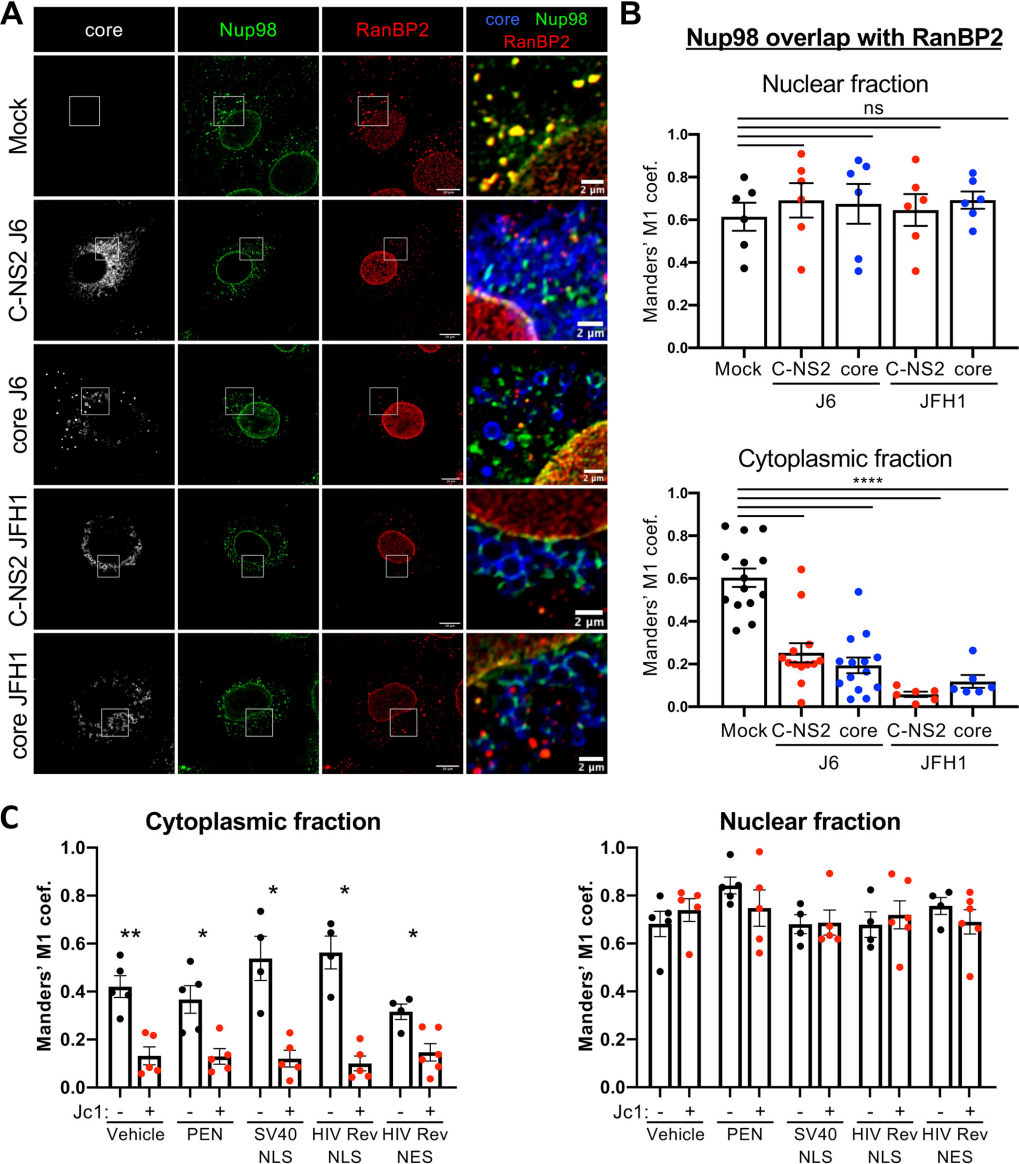
HCV core expression is sufficient to dissociate Nup98 from RanBP2 independently of karyopherins. (A and B) Huh7.5 cells were transduced with lentiviral vectors expressing core or core to NS2 (C-NS2) from J6 or JFH1 strains and were fixed at 72 h postransduction. (A) Nup98, HCV core, and RanBP2 proteins were immunostained, and the colocalization of Nup98 (green channel) with core (blue channel) and RanBP2 (red channel) was analyzed by confocal microscopy. Scale bars of panels and enlargements from the boxed area represent 10 μm and 2 μm, respectively. (B) The degree of overlap between nuclear or cytoplasmic Nup98 and RanBP2 was assessed by quantifying the Manders’ M1 overlap coefficients, representing the overlap of Nup98 signal with RanBP2 signals in the different fractions, with the JACoP plugin of ImageJ. (C) Huh7.5 cells were infected with Jc1 virus (MOI of 0.2) for 6 h and then incubated with 100 μM synthetic peptides targeting different karyopherins (25): PEN was used as a control, SV40 NLS binds to IPOA5 (importin alpha5), HIV-1 Rev NLS binds to IPO5 (importin beta3), and HIV-1 Rev NES binds to XPO1 (CRM1). At 72 h postinfection, cells were fixed and stained for core, Nup98, and RanBP2 proteins to assess the Nup98/RanBP2 colocalization by confocal microscopy. The degree of overlap between nuclear or cytoplasmic Nup98 and RanBP2 was assessed by quantifying the Manders’ M1 overlap coefficients, representing the overlap of Nup98 signal with RanBP2 signals in the different fractions, with the JACoP plugin of ImageJ.

Second, whereas Nup98 colocalized in Huh7.5 cells with RanBP2, we found that cytoplasmic Nup98 was readily disassociated from cytoplasmic RanBP2 in Jc1 and JFH1 HCVcc-infected cells, without any change in its nuclear fraction (Fig. 6C and D), and was readily redistributed to core-containing puncta. We obtained similar results of Nup98 dissociation from RanBP2-expressing AL, but not from TPR-expressing NPC, and relocalization to core-expressing structures upon individual expression of HCV core in Huh7.5 cells (Fig. 7A and B; see Fig. S9A and B in the supplemental material). Importantly, virus infection as well as core individual expression did not change the overall distribution of RanBP2 and TPR (i.e., at both the nuclear envelope and AL for RanBP2 versus exclusively at the nuclear envelope for TPR) (Fig. 6 and 7; Fig. S9), hence suggesting that HCV infection does not hijack functional or Nup98-located NPC, but rather seems to recruit Nup98 from annulate lamellae. Interestingly, we also observed that Nup98 was progressively excluded from cytosolic RanBP2-containing structures upon HCV infection (Fig. S9C), suggesting that the recruitment of Nup98 is highly correlated with assembly efficiency.

10.1128/mbio.02923-21.9 FIG S9 HCV core expression is sufficient to dissociate Nup98 from RanBP2. (A and B) Huh7.5 cells were transduced with lentiviral vectors expressing core or core to NS2 (C-NS2) from J6 or JFH1 strains and were fixed at 72 h posttransduction. (A) Nup98, HCV core, and TPR proteins were immunostained, and the colocalization of Nup98 (green channel) with core (blue channel) and TPR (red channel) was analyzed by confocal microscopy. (B) The degree of overlap between nuclear or cytoplasmic Nup98 and TPR was assessed by quantifying the Manders’ M1 overlap coefficients, representing the overlap of Nup98 signal with TPR signal in the different fractions, with the JACoP plugin of ImageJ. Scale bars of panels and enlargements from the boxed area represent 10 μm and 2 μm, respectively. (C) Huh7.5 cells were infected with Jc1 or JFH1 viruses (MOI of 0.2) and were fixed at 24, 48, or 72 h postinfection. HCV core, Nup98, and RanBP2 were immunostained, and the colocalization of Nup98 with RanBP2 was analyzed by confocal microscopy. The degree of overlap between nuclear or cytoplasmic Nup98 and RanBP2 was assessed by quantifying the Manders’ M1 overlap coefficients, representing the overlap of Nup98 signal with RanBP2 signal in the different fractions, with the JACoP plugin of ImageJ. Download FIG S9, PDF file, 1.7 MB. Copyright © 2022 Boson et al. 2022 Boson et al. https://creativecommons.org/licenses/by/4.0/ This content is distributed under the terms of the Creative Commons Attribution 4.0 International license.

Finally, since the core protein contains several nuclear localization signals (NLSs) and nuclear export signals (NESs) that may allow the binding of core to karyopherins (25) and since Nup98 contains a karyopherin-binding domain, we wondered if karyopherins may mediate recruitment of Nup98 by core. To test this hypothesis, we investigated the dissociation of Nup98 from AL in HCV- versus mock-infected cells in the presence of NLS- or of NES-containing peptides that can compete with core for binding to karyopherins (25). Interestingly, peptides previously shown to block the binding of HCV core to importin alpha5 (a simian virus 40 [SV40] NLS), importin beta3 (HIV-1 Rev NLS) or CRM1 (HIV-1 Rev NES) (25) could not prevent core-mediated dissociation of Nup98 from RanBP2 (Fig. 7C; see Fig. S10 in the supplemental material), hence suggesting that Nup98 is retrieved by core independently of karyopherins.

10.1128/mbio.02923-21.10 FIG S10 Effect of peptides targeting karyopherins on RanBP2-Nup98 dissociation induced by HCV infection. Huh7.5 cells were infected with Jc1 virus (MOI of 0.2) for 6 h and then incubated with 100 μM each synthetic peptides targeting different karyopherins (25): PEN was used as a control, SV40 NLS binds to IPOA5 (importin alpha5), HIV-1 Rev NLS binds to IPO5 (importin beta3), and HIV-1 Rev NES binds to XPO1 (CRM1). At 72 h postinfection, cells were fixed and stained for core (white channel), Nup98 (green channel), and RanBP2 (red channel) proteins to assess the Nup98/RanBP2 colocalization by confocal microscopy. Scale bars of panels and enlargements from the boxed area represent 10 μm and 2 μm, respectively. Download FIG S10, PDF file, 2.2 MB. Copyright © 2022 Boson et al. 2022 Boson et al. https://creativecommons.org/licenses/by/4.0/ This content is distributed under the terms of the Creative Commons Attribution 4.0 International license.

Altogether these results suggested that viral (+)RNA interacts with Nup98 in annulate lamellae and that core recruits Nup98 from these annulate lamellae to bring viral (+)RNA to HCV assembly sites.

## DISCUSSION

**FIG 8:**
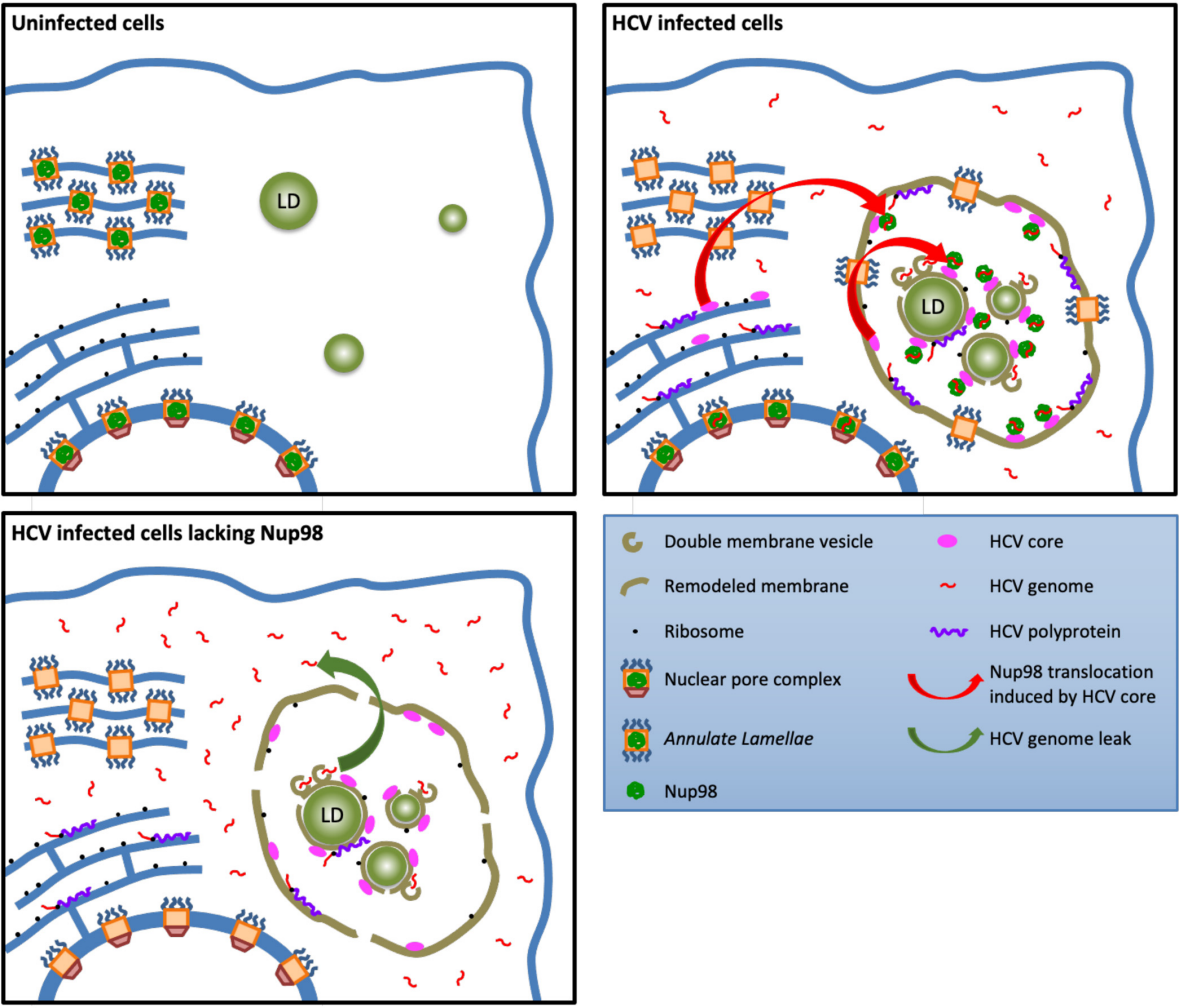
Working model. In uninfected Huh7.5 cells (top left panel), part of the nucleoporins is stored in cytoplasmic nuclear pore complex-like structures stacked in parallel ER membranes referred as annulate lamellae (AL). The infection of Huh7.5 cells by HCV (top right panel) induces the redistribution of AL to ER-derived remodeled membranes called membranous web (MW) (6), which is induced by HCV to achieve the viral replication, translation, and assembly within a protected environment. HCV core, probably through its high mobility on ER membranes (56), interacts with Nup98 from AL and translocates it within the MW and in close proximity of assembly sites, where it interacts with HCV RNA. In the absence of Nup98 (bottom left panel), the viral genome is excluded from the replication, assembly, and translation areas, leading to a decrease of infectious particle assembly. We propose that HCV hijacks Nup98, via its redistribution induced by core, to maintain the viral genome in the proper environment for its replication, assembly, and translation.

Our results underscore a novel mechanism by which a nucleoporin, Nup98, favors the early steps of assembly of HCV particles. Specifically, we found that (i) Nup98 interacts with viral RNA independently of virion assembly, (ii) core recruits Nup98 from annulate lamellae and increases Nup98-HCV (+)RNA apposition, and (iii) core-associated Nup98 maintains viral RNA in intracellular sites of HCV replication, translation, and assembly, in order to promote HCV assembly. Based on this evidence, we propose that annulate lamella (AL) pore complexes (ALPCs) could be found in the “membranous web” (MW) and allow the retention of viral RNA in this specific microenvironment where HCV replication, translation, and assembly occur. Thus, when the replication to assembly switch occurs after a certain threshold of expression of viral assembly components is reached, core recruits Nup98 from these AL to favor encapsidation of viral ribonucleoprotein in nascent particles (see working model in Fig. 8).

### Role of Nup98 in HCV infection.

HCV infection induces a marked rearrangement of ER membranes, hence creating the so-called membranous web (20, 21). This specific area is believed to favor HCV RNA replication and virion assembly in a protected environment that may also prevent activation of innate immunity (26). Here, we show that Nup98 can maintain viral genomes in areas where replication, translation, and assembly occur (Fig. 8). Noteworthy, a recent study indicated that components from the nuclear pore complex (NPC), including Nup98, could be recruited and form “doors” at MW boundaries (6). It was proposed that such doors may control the selective transport of larger molecules, such as proteins harboring nuclear localization or export signals (NLSs/NESs), which could preclude access of immune receptors to this compartment, while allowing the passive diffusion of small molecules required for HCV replication (7). Remarkably, our results highlight that Nup98 acts as a key component that prevents the escape of viral RNAs from replication and assembly areas and that is recruited by HCV core. Indeed, Nup98 harbors an RNA-binding domain (16), which may allow the capture of HCV (+)RNAs after their synthesis during replication. Furthermore, a recent proteomic study indicated that HCV core can interact with Nup98 in HCV-infected cells (5).

We foresee different roles for such a triple HCV (+)RNA-core-Nup98 interaction. First, while it may enrich the concentration of HCV (+)RNAs in MW areas, which are specifically depleted from immune sensors (7), it may also reduce the concentration of (+)RNAs in the MW surrounding regions where immunity receptors are located, hence contributing to reduce activation of innate immune pathways. Another possibility is that Nup98 could stabilize either viral RNAs alone or (+)RNA-core complexes to protect them from degradation. Third, we propose that, like for canonical NPCs, Nup98-containing ALPCs could allow the unrestricted diffusion of viral RNA through this pore, in agreement with its localization throughout the cytosol (Fig. 6C and D); yet, when the core expression level reaches a certain threshold, it could hijack Nup98 from the ALPCs in order to induce retention of viral RNAs in the MW and more precisely in close proximity to assembly factors (Fig. 3C and D).

In addition to allowing maintenance of viral (+)RNAs within the MW, our results open the possibility that core hijacks Nup98 to bring the viral genome close to the assembly sites, hence promoting the initiation of its encapsidation in nascent virions. Of note, no specific packaging determinant has been identified in HCV RNA to date (27), which has raised the question of how HCV targets its genomic RNAs to assembly sites. Interestingly, the core protein contains several nuclear localization signals (NLSs) (25) with a poorly defined role, although these NLSs are located in the RNA binding region of core (28). Despite that these NLSs may allow the binding of core to karyopherins (25) and that Nup98 contains both an RNA-binding domain and a binding domain to karyopherins, we showed that core recruits Nup98 independently of karyopherins (Fig. 7C). Moreover, our data suggest that viral RNA-loaded Nup98 could be recruited by core only upon a replication-to-assembly switch, which could match with the stabilization of core within infected cells (29). Interestingly, Nup98 was shown to be involved in trafficking within the nucleus to bring cellular RNAs close to NPCs (30); thus, we propose a similar viral RNA trafficking mediated by Nup98 within the MW. Overall, it is possible that the triple (+)RNA-Nup98-core interaction would form the HCV RNA packaging determinant. In this respect, via its interaction with RNAs, Nup98 could be essential for assembly (Fig. 1) by maintaining a high concentration of viral RNAs close to the core and assembly site. This possibility may also agree with the presence of Nup98 within particles, as shown by proteomic analysis (5).

### Unique mechanism of perversion of nuclear pore complex components by HCV.

Several RNA viruses have been shown to hijack NPCs; yet, to the best of our knowledge, none of them seem to use nucleoporins and/or NPC components in a manner similar to that underscored here for HCV. Indeed, our results highlight an original mechanism whereby HCV hijacks a cytosolic nucleoporin to promote its replication and assembly, whereas most other viruses directly act on NPC to facilitate viral replication and/or to inhibit innate immunity. On the one hand, several viruses, such as, e.g., influenza A virus (31), vesicular stomatitis virus (32, 33), and human immunodeficiency virus (34), disrupt host mRNA nuclear export pathways, which decreases the competition between host and viral RNAs for translation and inhibits the expression of antiviral factors. On the other hand, some other viruses, such as, e.g., Ebola virus (35, 36), poliovirus, and human rhinovirus (37–40), or severe acute respiratory syndrome coronavirus (SARS-CoV) (41) and SARS-CoV-2 (42, 43) disrupt the nucleocytoplasmic trafficking of proteins, through different mechanisms, including binding to karyopherins or cleavage of Nups (reviewed in reference 34), hence blocking nuclear translocation of important cellular proteins involved in immunity.

Our study does not exclude that HCV could also act on NPCs like for these other viruses. However, arguing against a specific impairment of nuclear transport, HCV infection was shown to increase the levels of some nucleoporins, including Nup88, Nup214, Nup358, and Nup153 (6), in contrast to other *Flaviviridae* members that decrease nucleoporin levels (44). This is in agreement with our results and highlights a specific feature of HCV indicating that this virus uses nucleoporins as proviral factors for replication and assembly.

### Annulate lamellae: poorly characterized membrane organelles in viral infection.

Annulate lamellae are cytoplasmic membrane cisternae that are continuous with the ER (22). ALPCs are inserted into these membranes, although, in contrast to NPCs, they are symmetric, with both of their sides open to the cytosol (45). Interestingly, most NPC nucleoproteins are also present in ALPCs; yet, some NPC components, especially those of the nuclear pore basket like TPR, are absent from ALPCs (46). The precise roles of annulate lamellae and ALPCs remain unknown, although it was proposed that they represent “membrane and NPC” reservoirs that could be used for nuclear expansion after cell division and nuclear membrane reformation (47).

The role of these membranes and ALPCs in viral infections remains poorly studied, mainly because they are difficult to identify. Indeed, electron microscopy (EM) is the “gold standard” method to firmly identify these particular structures, but due to the thickness of the EM samples, they must be perfectly in plane to be correctly visualized. Besides, fluorescence microscopy is a method of choice to link AL visualization with functional analysis. Indeed, recent studies have shed light on AL composition (10, 23, 24), allowing us to detect them by fluorescence microscopy. The number of AL seems to be increased upon infection by several viruses, including hepatitis A virus (48), Japanese encephalitis virus (49), and SARS-CoV-2 (50), or appears to localize at close proximity to remodeled membranes induced by HCV (12); yet, their precise role was not characterized in these studies. It has also been proposed that AL could represent storage vesicles for viral glycoproteins in the case of human herpesvirus 6 (51). Together with the results of others (6, 7), our report suggests that HCV may use AL, or at least specific ALPC components such as Nup98, to provide a source of membranes with functional pores, which may facilitate the establishment of its MW and the selective transport of molecules. This is likely to be crucial for this virus in order to create a protected environment for replication that can gain controlled access to the entire cytoplasm, as discussed above.

Collectively, our results provide new insights into the cellular factors and organelles that promote HCV assembly. Specifically, we show that Nup98 could interact with viral RNA in the annulate lamella pore complex upon replication. When assembly of viral particles takes place, core may recruit Nup98 to allow the initiation of encapsidation of viral RNA. Overall, this highlights how HCV could hijack and subvert some cellular components for its own benefit.

## MATERIALS AND METHODS

### Cell culture and reagents.

Huh7.5 cells (a kind gift from Charles Rice, Rockefeller University, USA) and HEK-293T cells (ATCC CRL-1573) were grown in Dulbecco’s modified minimal essential medium (DMEM) (Invitrogen, France) supplemented with 100 U/mL of penicillin, 100 μg/mL of streptomycin, and 10% fetal bovine serum.

### Expression constructs.

pFK-JFH1wt_dg, pFK-JFH1/J6/C-846_dg, and pWPI-CNS2 Jc1 plasmids (52, 53) were kind gifts from R. Bartenschlager (Heidelberg University, Germany). For the pWPI-CNS2 JFH1 construct, the fragment encompassing the beginning of core to the middle of NS2 was extracted from the JFH1 C-NS2 construct (17) with BamHI and NheI restriction enzymes and cloned into pWPI-CNS2 Jc1 opened with the corresponding enzymes. For pWPI-core J6 and pWPI-core JFH1 constructs, the entire core sequences were extracted from phCMV-IRES J6 core or phCMV-IRES JFH1 core constructs (17) with BamHI and SpeI restriction enzymes and cloned into pWPI-CNS2 Jc1 opened with the corresponding enzymes.

The pFlagNup98-sh5res plasmid was constructed as follows: The pEGFP-Nup98 plasmid was obtained from Jan Ellenberg (EMBL Heidelberg, Germany) (54). The N-terminal enhanced green fluorescent protein (EGFP) coding sequence was removed and replaced by a Flag tag coding sequence by mutagenesis with the Up Flag-Nup98 AgeI-HindIII and Low Nup98 StuI primers to generate the pFlagNup98 plasmid. The nucleotide sequence targeted by the sh5 shRNA was then modified by mutagenesis with the FwNup98_StuI and RevNup98_AgeI_sh5Res primers to introduce several silent mutations conferring to this construct the resistance of the downregulation of Nup98 by sh5 shRNA vector.

The NanoLuc luciferase gene was amplified from pL-Luc (a kind gift from Eric Bergeron) with the Up nLuc BamHI and Low nLuc XhoI primers and inserted in the pHR′-CMV-W-GFP (a kind gift from Didier Trono) lentiviral construct opened with BamHI and XhoI restriction enzymes to generate the pHR′-CMV-nLuc-WPRE lentiviral construct.

pSGR-JFH1-neoR is a kind gift from Franck Chisari. psPAX2 and phCMV-G are kind gifts from Didier Trono and Jane Burns, respectively. Lentiviral vectors encoding shRNA against Nup98 (TRCN0000046913 [sh1], TRCN0000046914 [sh2], TRCN0000046916 [sh3], TRCN0000296791 [sh4], TRCN0000296863 [sh5]), and SHC001 (pLKO.1 control empty vector) were purchased from Sigma-Aldrich. The puromycin resistance gene and its PGK promoter were partially deleted from SHC001 and TRCN0000296863 plasmids by extracting the BspEI fragment to generate SHC001-puro(−) and TRCN0000296863-puro(−), respectively. These plasmids were then used in ribopuromycylation experiments only.

### Antibodies and reagents.

Rat anti-Nup98 (clone 2H10), rabbit anti-TPR, and rabbit anti-RanBP2 were purchased from Abcam. Rabbit anti-Flag, mouse anti-PMY (clone 12D10), and mouse anti-β-actin (clone AC74) were purchased from Sigma-Aldrich. The mouse anti-nanoLuc antibody (MAB10026) was purchased from R&D Systems. Rat anti-E2 clone 3/11, mouse anti-NS5A clone 9E10, and mouse anti-core clone 19D9D6 were kind gifts from Jane McKeating (Oxford University, United Kingdom), Charles Rice (Rockefeller University, USA), and Colette Jolivet (bioMérieux, France), respectively.

BODIPY 493/503, ProLong glass antifade mountant, Hoechst 33342, and protein G Dynabeads were purchased from Thermo Fisher. Mowiol 40-88, cycloheximide (CHX), and puromycin were purchased from Sigma-Aldrich.

### Generation of subgenomic replicon cell line.

XbaI-linearized pSGR-JFH1-neoR DNA was *in vitro* transcribed with a T7 RiboMAX Express Large Scale RNA Production System kit (Promega), and 10 μg of the *in vitro*-transcribed RNA was electroporated on Huh7.5 cells. At 48 h after electroporation, the cells were cultured under 0.25 mg/mL of neomycin until a uniform layer of resistant cells appeared. The resistant cell population was further validated for NS5A expression by IF and flow cytometry.

### HCVcc production and titration.

HCVcc production procedures were described previously (17). Extracellular and intracellular infectivity titers were determined as focus-forming units (FFU)/mL. Serial dilutions of supernatants were used to infect Huh7.5 cells, FFU were determined 3 days postinfection by counting NS5A-immunostained foci.

### Production of lentiviral vectors.

A total of 2.4 × 10^6^ HEK293T cells were transfected with 2 μg of phCMV-G, 8 μg of psPAX2, and 8 μg of pWPI-CNS2 Jc1, pWPI-CNS2 JFH1, pWPI-core J6, pWPI-core JFH1, pHR′-CMV-nLuc-WPRE, TRCN0000046913, TRCN0000046914, TRCN0000046916, TRCN0000296791, TRCN0000296863, TRCN0000296863-puro(−), SHC001, or SHC001-puro(−) plasmids using calcium phosphate precipitation. At 16 h posttransfection, the medium was changed and harvested 24 h later, filtered through 0.45-μm-pore filters, and stored aliquoted at −80°C. Lentiviral vectors expressing HCV proteins or nLuc were titrated on Huh7.5 cells. Briefly, 10^5^ Huh7.5 cells were transduced with serial dilutions of the lentiviral vectors, and the expression of the HCV core or nLuc was analyzed 72 h later by flow cytometry.

### Downregulation of Nup98.

A total of 1 × 10^5^ cells were seeded on 6-well plates containing uncoated 14-mm-diameter glass coverslips. Eight hours later, cells were transduced with lentiviral vectors expressing a control shRNA (SHC001) or an shRNA against Nup98 (TRCN0000046913 [sh1], TRCN0000046914 [sh2], TRCN0000046916 [sh3], TRCN0000296791 [sh4], or TRCN0000296863 [sh5]). The sh5 shRNA, which conferred the best downregulation of Nup98, was used in all experiments and referenced as shNup98, with the exception of Fig. S1A and B, in which all shRNAs were tested. Sixteen hours postransduction, cells were washed and infected with Jc1 or JFH1 viruses at a multiplicity of infection (MOI) of 0.2. For gain-of-function experiments, Nup98-downregulated Huh7.5 cells were transfected with pFlagNup98-sh5res or a GFP-expressing construct as a control with GeneJammer (Agilent) according to the manufacturer’s instructions prior to infection with Jc1 or JFH1 viruses. When stated, cells were also transduced with lentiviral vector expressing nLuc at the same time with an MOI of 10. Six hours later, cells were washed with fresh medium and cultured for the indicated time before fixation.

### Immunoprecipitation.

A total of 1.2 × 10^6^ Huh7.5 or SGR cells were infected with Jc1 at an MOI of 0.5 or transduced with CNS2 Jc1-expressing lentiviral vectors at an MOI of 5, respectively. For experiments with nLuc vector, 1.2 × 10^6^ Huh7.5 cells were transduced with nLuc-expressing lentiviral vector at an MOI of 10. Six days postinfection or transduction, 4 × 10^6^ cells were washed and lysed at 4°C for 30 min in lysis buffer (50 mM Tris HCl [pH 7.5], 50 mM NaCl, 0.2% bovine serum albumin [BSA], 0.5% Triton X-100, mini-EDTA-free protease inhibitor mixture [Roche]). Lysates were clarified at 18,000 × *g* at 4°C for 15 min and incubated overnight with 50 μL of protein G Dynabeads coated with 5 μg of anti-Nup98 or anti-rat IgG. Immune complexes were washed 3 times with washing buffer 1 (50 mM Tris HCl [pH 7.5], 50 mM NaCl, 0.1% BSA, 0.25% Triton X-100) and twice with washing buffer 2 (50 mM Tris HCl [pH 7.5], 50 mM NaCl) and eluted with Laemmli buffer for 5 min at 95°C before Western blot analysis or RNA quantification.

### Western blot analysis.

Proteins obtained in total cell lysates or by immunoprecipitation were denatured in Laemmli buffer at 95°C for 5 min, separated by SDS-PAGE, and then transferred to nitrocellulose membrane and revealed with specific primary antibodies, followed by the addition of IRdye secondary antibodies (goat anti-Rat IRDye 680RD, donkey anti-mouse IRDye 680RD, and donkey anti-rabbit IRDye 800CW; Li-Cor Biosciences), and analyzed by imaging with an Odyssey infrared imaging system CLx (Li-Cor Biosciences).

### RNA quantifications.

HCV RNAs were extracted (TRI reagent; Euromedex), reverse transcribed (iScript cDNA synthesis kit; Bio-Rad), and quantified (FastStart Universal SYBR green master kit; Roche Applied Science) on an Applied StepOne real-time PCR apparatus. The sequences of the primers used for the reverse transcription-quantitative PCR (RT-qPCR) were as follows: for HCV, 5′-TCTGCGGAACCGGTGAGTA-3′ and 5′-TCAGGCAGTACCACAAGGC-3′; for glyceraldehyde-3-phosphate dehydrogenase (GAPDH), 5′-AGGTGAAGGTCGGAGTCAACG-3′ and 5′-TGGAAGATGGTGATGGGATTTC-3′; for transferrin (TF), 5′-CCCTTAACCAATACTTCGGCTAC-3′ and 5′-TTTGCCAAGTTCTCAAATATAGTCG-3′; for actin, 5′-TCCGTGTGGATCGGCGGCTCCA-3′ and 5′-CTGCTTGCTGATCCACATCTG-3′; for CIDEB, 5′-AGCCAAAGCATTGGAGACCCTACT-3′ and 5′-TCTGACCAGACTGCAACACCATCA-3′; and for HIV vector (nLuc), 5′-TGTGTGCCCGTCTGTTGTGT-3′ and 5′-GAGTCCTGCGTCGAGAGAGC-3′. RNA levels were normalized with respect to GAPDH RNA levels. As an internal control of extraction, an exogenous RNA from the linearized Triplescript plasmid pTRI-Xef (Invitrogen) was added into the supernatant prior to extraction and quantified with specific primers (5′-CGACGTTGTCACCGGGCACG and 5′-ACCAGGCATGGTGGTTACCTTTGC).

### Competition assay with peptides targeting karyopherins.

Synthetic peptides were manufactured by GenScript (Netherlands) and have been previously described (25). PEN was used as a control, SV40 NLS binds to IPOA5 (importin α5), HIV-1 Rev NLS binds to IPO5 (importin β3), and HIV-1 Rev NES binds to XPO1 (CRM1). Huh7.5 cells grown on uncoated 14-mm-diameter glass coverslips were infected at an MOI of 0.2 for 6 h. The virus was then washed and replaced by fresh DMEM containing 100 μM each peptide or vehicle (H_2_O). At 72 h postinfection, cells were washed with PBS, fixed with 3% paraformaldehyde (PFA), and further stained for core, Nup98, and RanBP2 proteins to assess the Nup98/RanBP2 colocalization by confocal microscopy.

### Immunofluorescence and confocal microscopy imaging.

Huh7.5 cells grown on uncoated 14-mm-diameter glass coverslips were infected at MOI of 0.2. At different times postinfection, cells were washed with PBS, fixed with 3% paraformaldehyde in PBS for 15 min, quenched with 50 mM NH_4_Cl, and permeabilized with 0.1% Triton X-100 for 7 min. Fixed cells were then incubated for 1 h with primary antibodies in 1% BSA–PBS, washed and stained for 1 h with the corresponding fluorescent Alexa Fluor-conjugated secondary antibody (Alexa Fluor 488, Alexa Fluor 568, and Alexa Fluor 647; Thermo Fisher) in 1% BSA–PBS. LDs were stained with 10 μg/mL BODIPY 493/503 according to the manufacturer’s instructions. Cells were washed three times with PBS, stained for nuclei with Hoechst for 5 min when stated, washed and mounted in Mowiol prior to image acquisition with LSM-710 confocal microscope.

### Combined detection of HCV RNA by fluorescent *in situ* hybridization and viral proteins.

Viral proteins were first immunostained as described above. After a postfixation step with 3% paraformaldehyde for 15 min and 3 washes with PBS, HCV RNA plus and minus strands were detected using probe sets that target regions between nucleotide positions 3733 to 4870 and 4904 to 5911, respectively (VF1-10121 and VF6-11102; Affymetrix). In the JFH1 genome, the nLuc RNA was detected using a probe that targets the full WPRE sequence contained in the lentiviral genome sequence (VF4-6001192; Affymetrix), and actin and GAPDH RNAs were detected with the control probes VA4-10293 and VA4-10641, respectively (Affymetrix), using the QuantiGene ViewRNA ISH cell assay kit (Panomics/Affymetrix) according to the manufacturer’s instructions, except for the protease digestion step, which was omitted. Staining of nuclei, slide mounting, and acquisition were performed as described above.

### Ribopuromycylation assay.

A total of 10^5^ Huh7.5 cells were seeded on 6-well plates containing uncoated 14-mm-diameter glass coverslips. Eight hours postseeding, cells were transduced with lentiviral vectors expressing a control shRNA [SHC001-puro(−)] or an shRNA against Nup98 [TRCN0000296863-puro(−)]. Sixteen hours postransduction, cells were infected with Jc1 or JFH1 viruses at MOI of 0.2. At 72 h postinfection, cells were processed for ribopuromycylation as previously described (14). Briefly, cells were incubated with 355 μM cycloheximide (CHX) and 91 μM puromycin or with cycloheximide alone as control for 5 min at 37°C. Cells were then put on ice and washed once with ice-cold PBS. Nonincorporated puromycin was then removed by incubating the cells for 2 min on ice with ice-cold extraction buffer (0.015% digitonin, 50 mM Tris-HCl [pH 7.5], 5 mM MgCl_2_, 25 mM KCl, 355 μM CHX, mini-EDTA-free protease inhibitor mixture). The cells were then washed once with wash buffer (50 mM Tris-HCl [pH 7.5], 5 mM MgCl_2_, 25 mM KCl, 355 μM CHX, mini-EDTA-free protease inhibitor mixture), fixed with 3% paraformaldehyde for 15 min at room temperature, and processed for IF and FISH.

### Structured illumination microscopy.

Huh7.5 cells were grown on high-precision cover glasses and infected (MOI of 0.2). At 72 h postinfection, cells were fixed with 3% paraformaldehyde and stained as described above. Cells were then mounted in ProLong glass antifade mountant prior to image acquisition with Elyra PS-1 microscope. Images were reconstructed using the Zen 2012 Black software.

### Image analysis and quantifications.

Images were analyzed with the ImageJ software. For quantifications of structures, colocalized pixels were extracted with the ColocalizeRGB plugin with autothresholding and a pixel ratio between paired channels set to 50%. They were then segmented with the Watershed algorithm and quantified with the “Analyze Particles” function of ImageJ. Only structures with a size of >0.02 μm^2^, which corresponds to half of the resolution limit of confocal laser scanning microscopy, were recorded. For quantification of Nup98 apposed at the edge of LDs or apposed to HCV (+)RNA, we used an ImageJ macro that we previously described (13) to search for variation of maximum intensity in the neighborhood of each LD or (+)RNA as an indicator of proximity. Nup98 structures distant from LDs or HCV (+)RNA by more than 3 pixels (ca. 205 to 207nm) were considered nonapposed. When stated, the Pearson’s correlation or Manders’ M1 coefficients were calculated by using the JACoP plugin. For Manders’ M1 coefficients, the nucleus was first segmented according to the Hoechst staining, and the quantifications were done inside or outside the nucleus region, representing the nuclear fraction or the cytoplasmic fraction, respectively. For the segmentation analysis, a Gaussian blur filter with a sigma of 10 pixels was applied to the images followed by a Huang autothresholding. The different parameters were then recorded inside or outside the segmented areas with the “Analyze Particles” function of ImageJ.

### Statistical analysis.

Significance values were calculated by applying the two-tailed, unpaired Mann-Whitney test for image analyses and the paired *t* test for the quantification of viral RNA, protein expression, and infection assays, using the GraphPad Prism 8 software (GraphPad Software, USA). *P* values of <0.05 were considered statistically significant, and the following denotations were used: ****, *P* ≤ 0.0001; ***, *P* ≤ 0.001; **, *P* ≤ 0.01; *, *P* ≤ 0.05; ns, not significant (*P* > 0.05).
