# Supplementary figures and images for: Nup98 Is Subverted from Annulate Lamellae by Hepatitis C Virus Core Protein to Foster Viral Assembly
Source: mBio. 2022 Mar 8;13(2):e02923-21. doi: 10.1128/mbio.02923-21 (PMC9040885; doi:10.1128/mbio.02923-21)

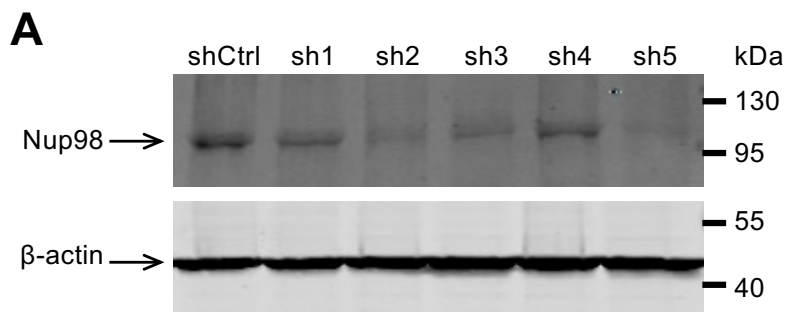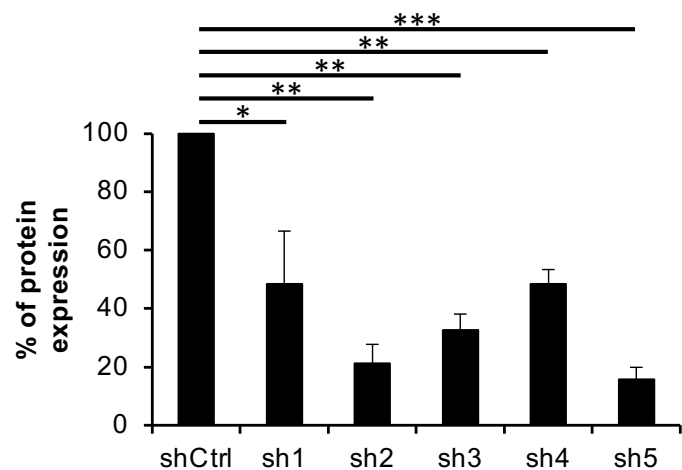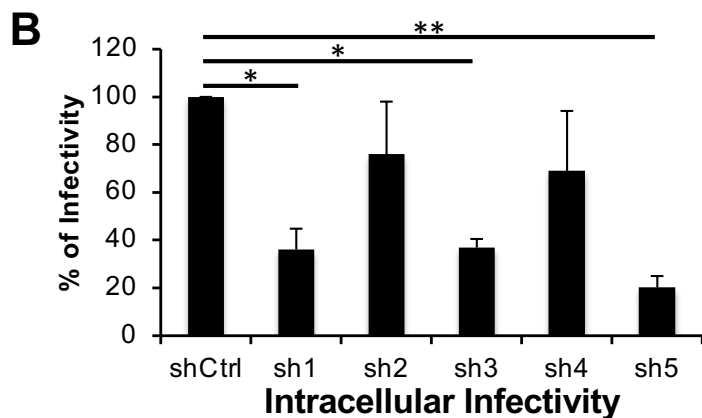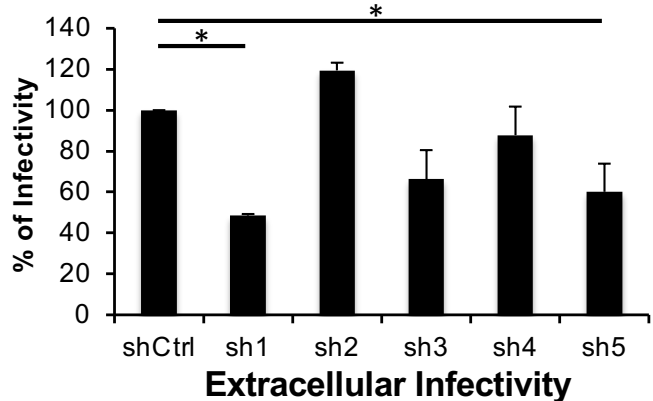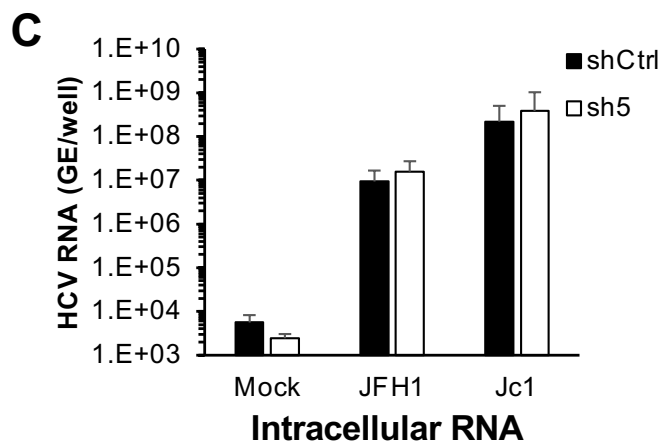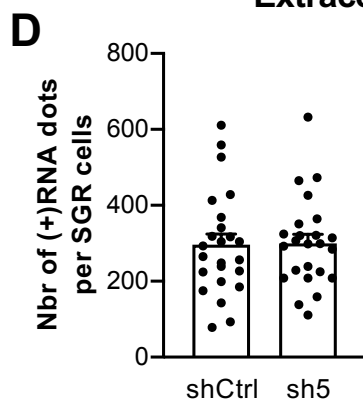

Supplemental Figure 1. Boson et al.

Supplement: FIG S1 [file mbio.02923-21-sf001.pdf]

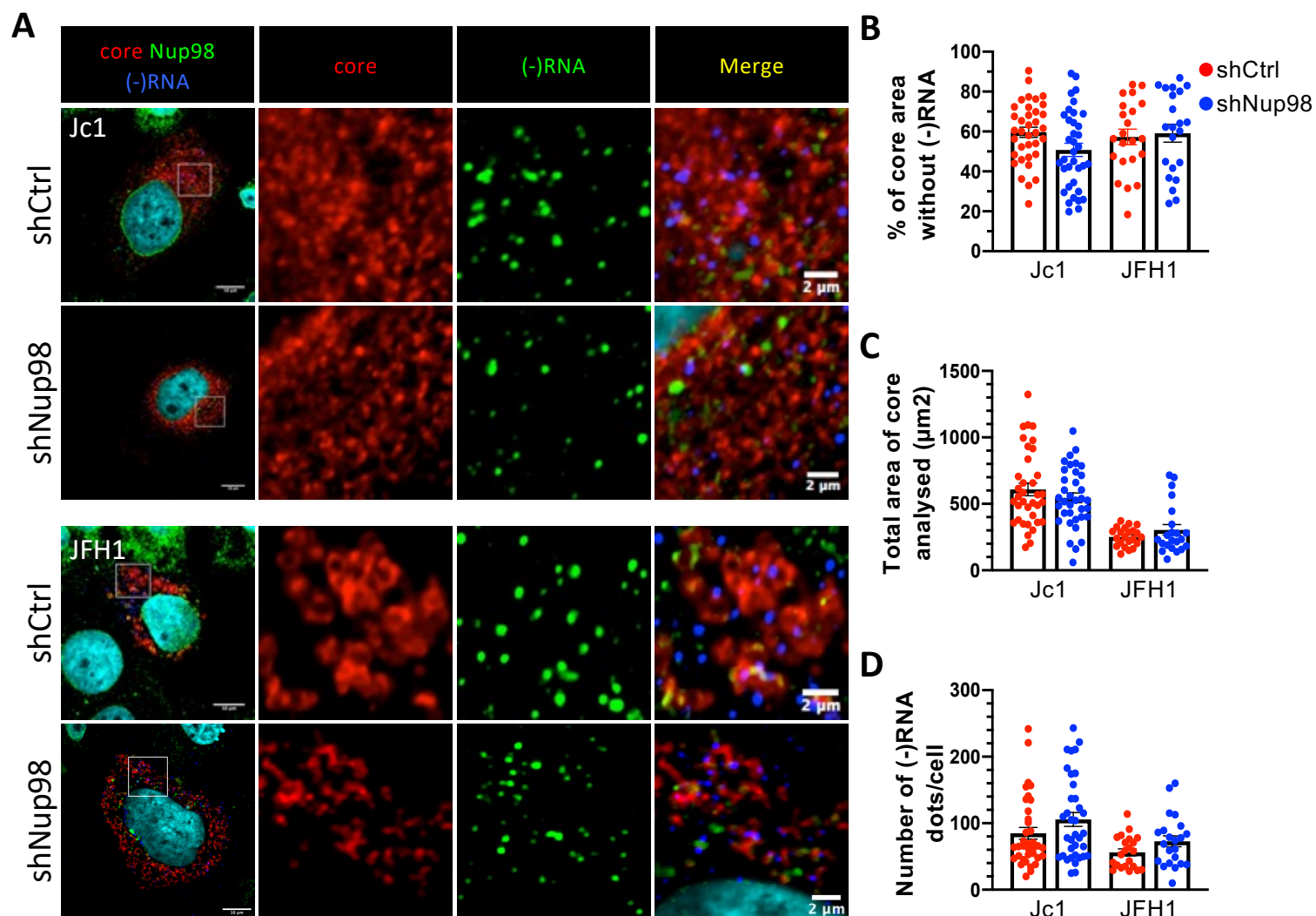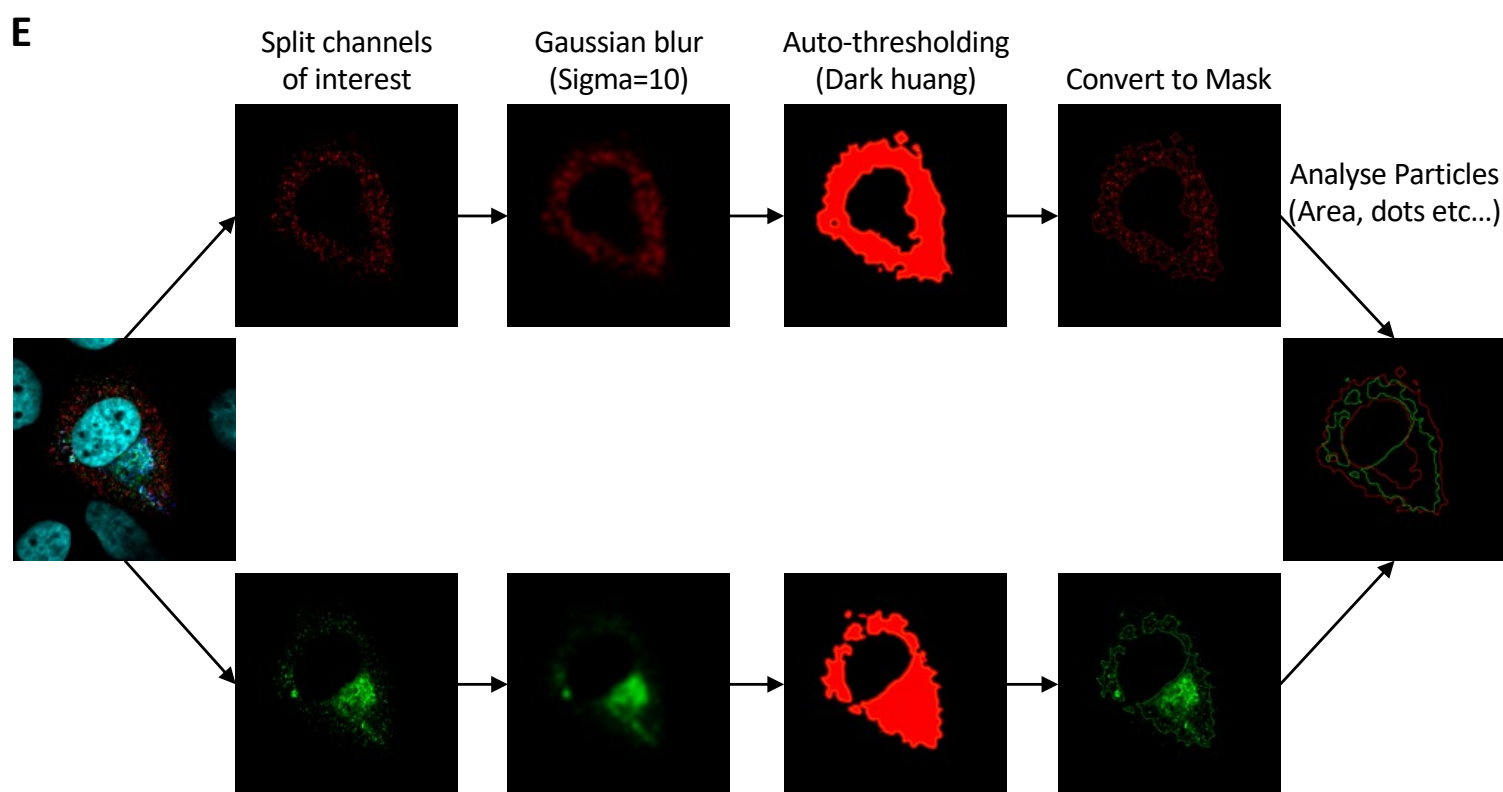

Supplemental Figure 2. Boson et al.

Supplement: FIG S2 [file mbio.02923-21-sf002.pdf]

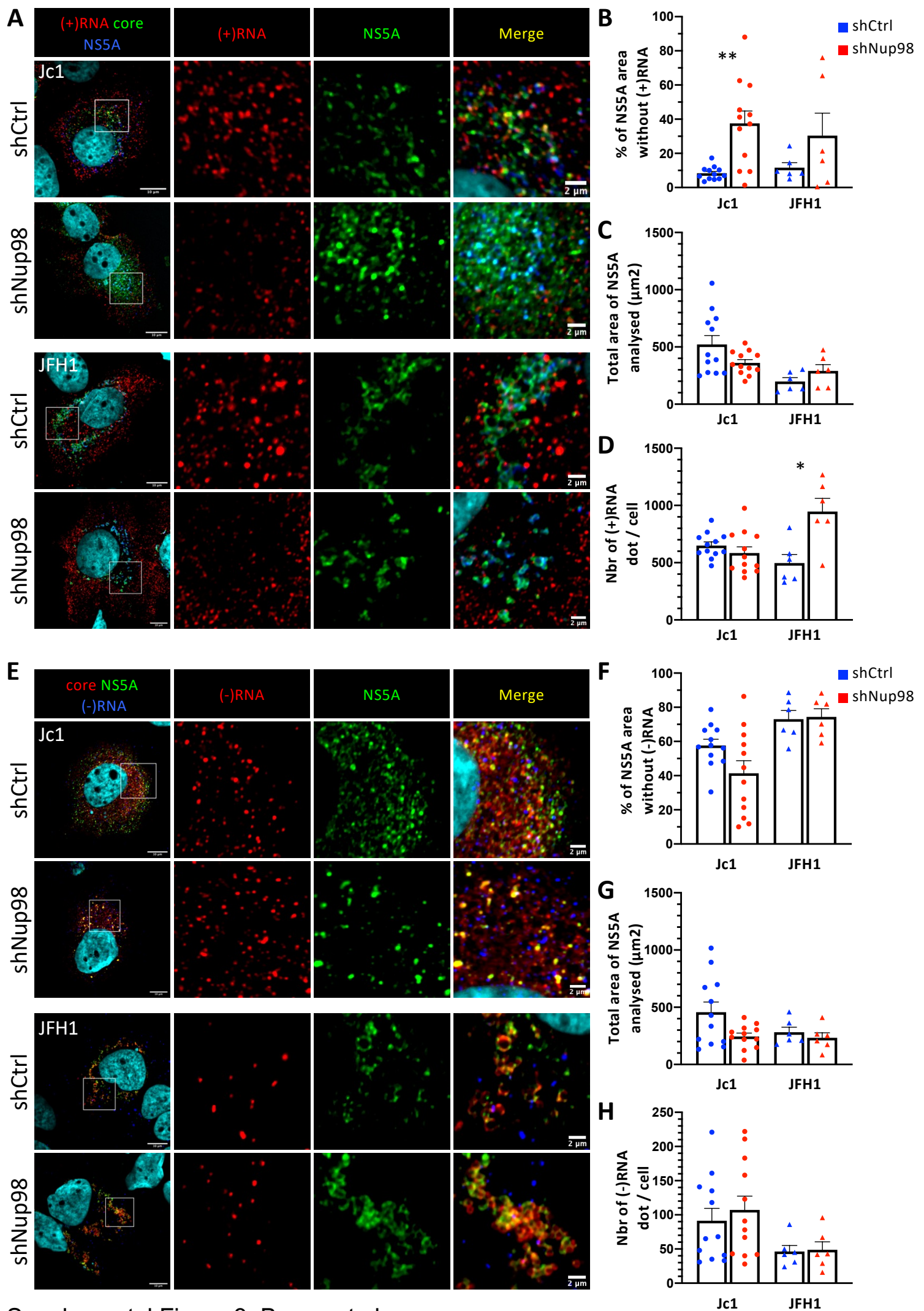

Supplemental Figure 3. Boson et al.

Supplement: FIG S3 [file mbio.02923-21-sf003.pdf]

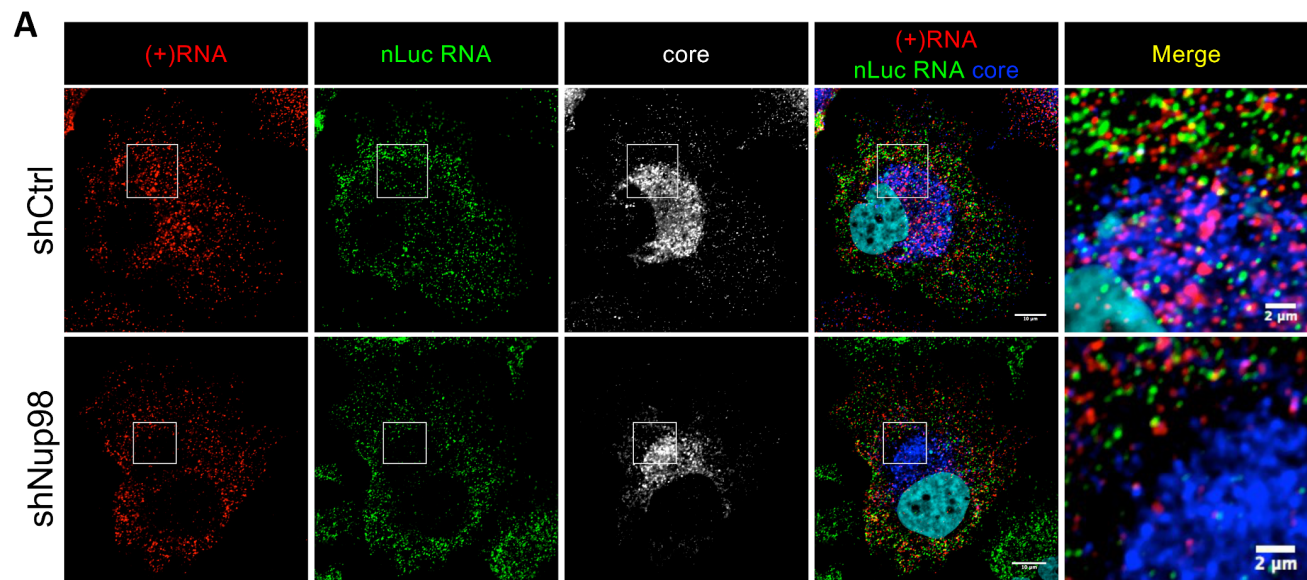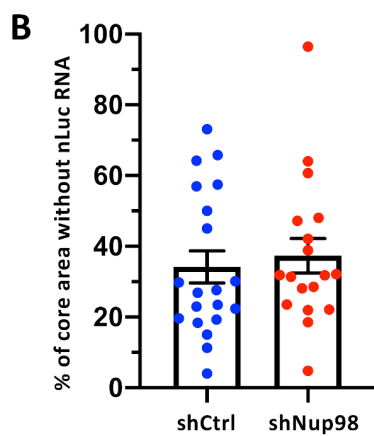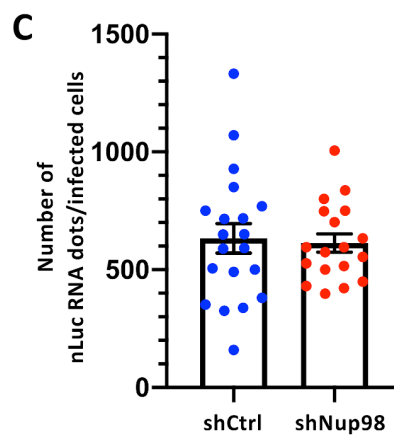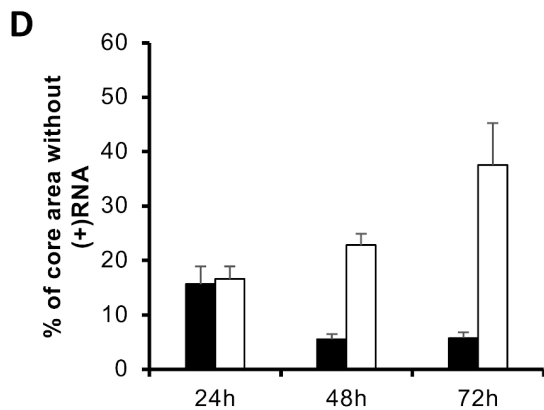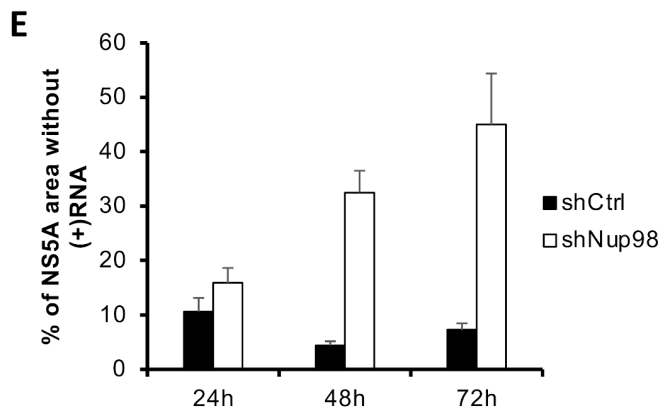

Supplemental Figure 4. Boson et al.

Supplement: FIG S4 [file mbio.02923-21-sf004.pdf]

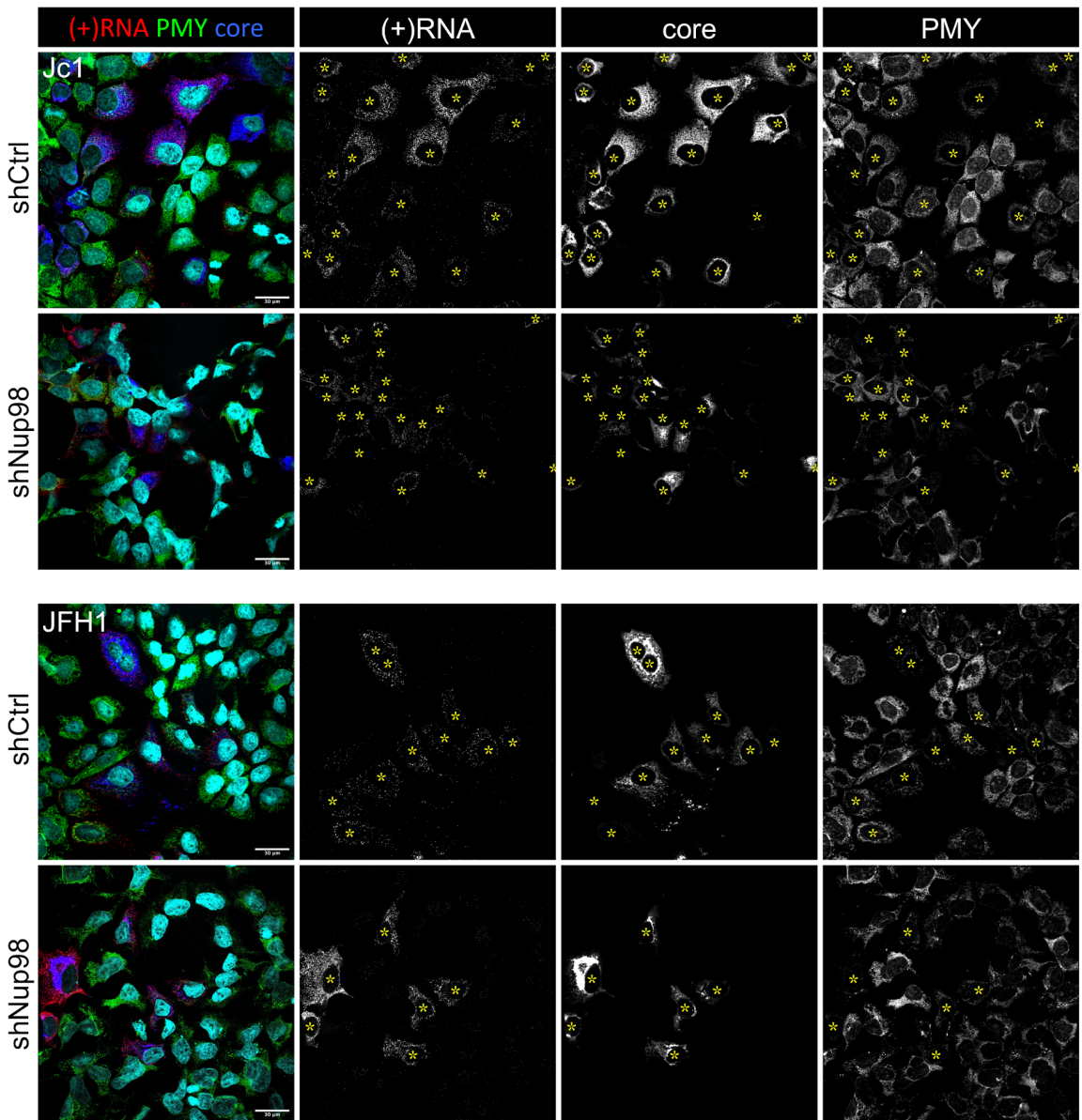

Supplemental Figure 5. Boson et al.

Supplement: FIG S5 [file mbio.02923-21-sf005.pdf]

**A**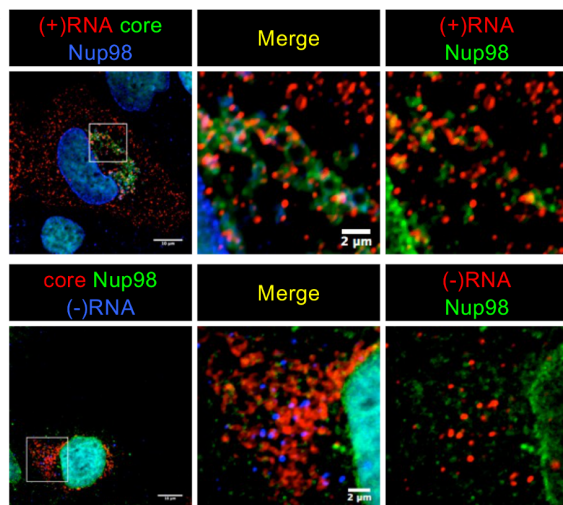**B**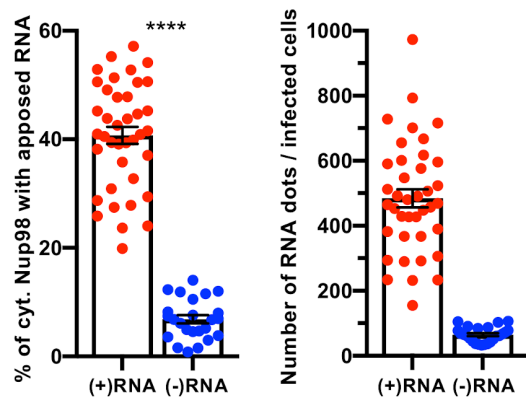**C**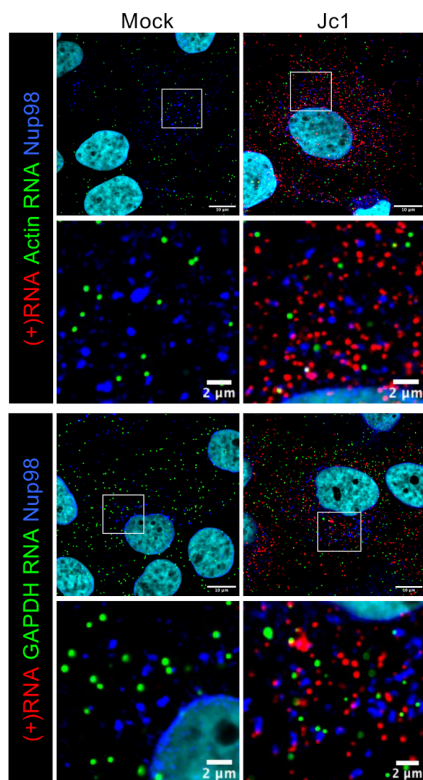**D**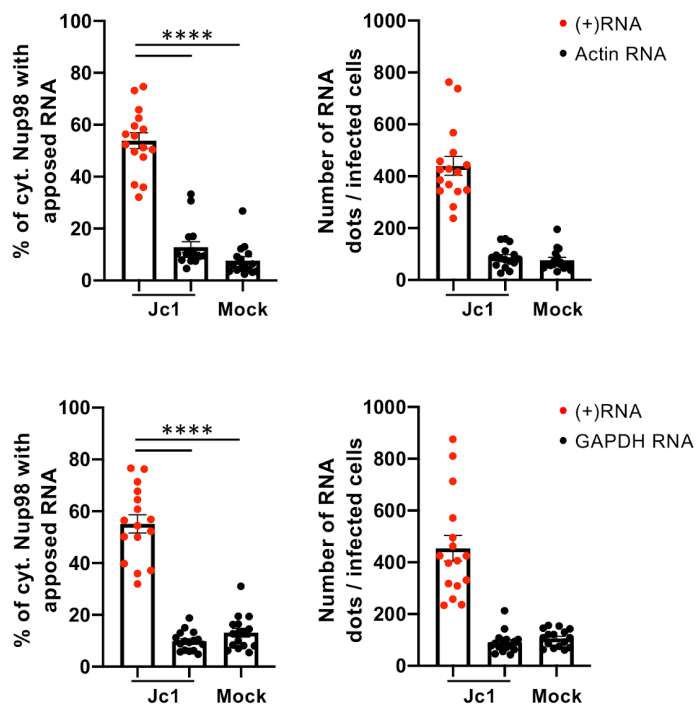**E**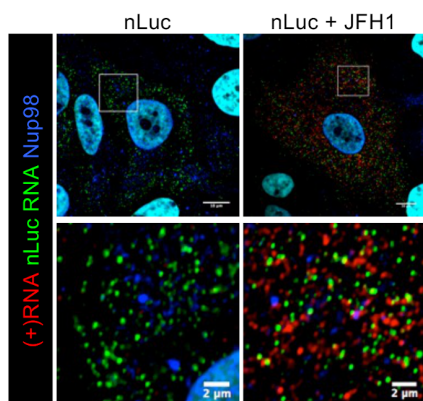**F**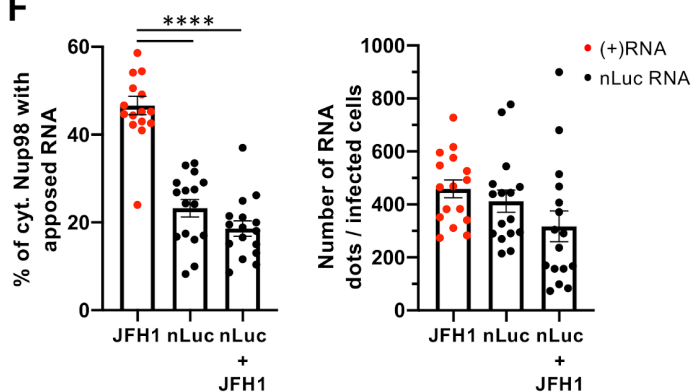

Supplement: FIG S6 [file mbio.02923-21-sf006.pdf]

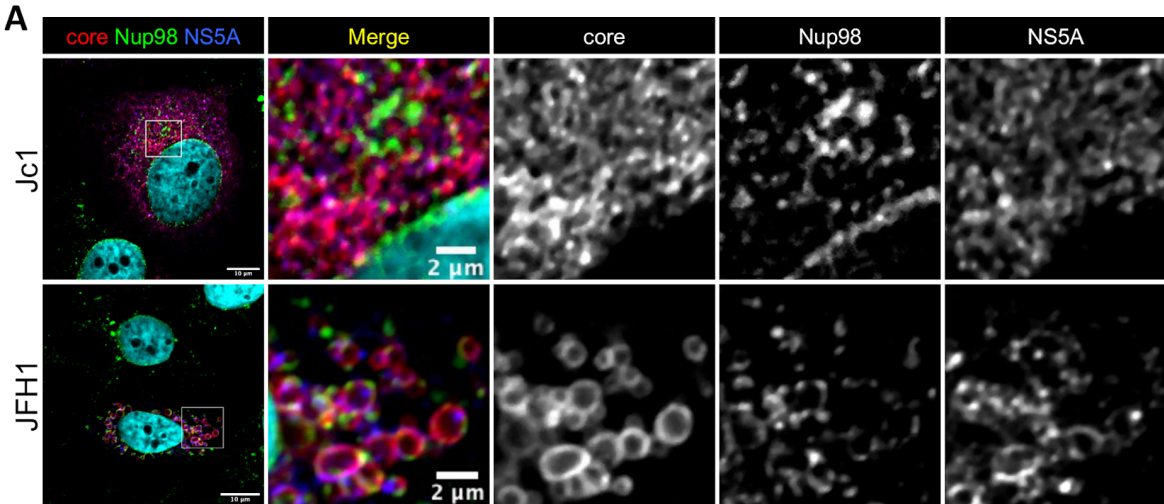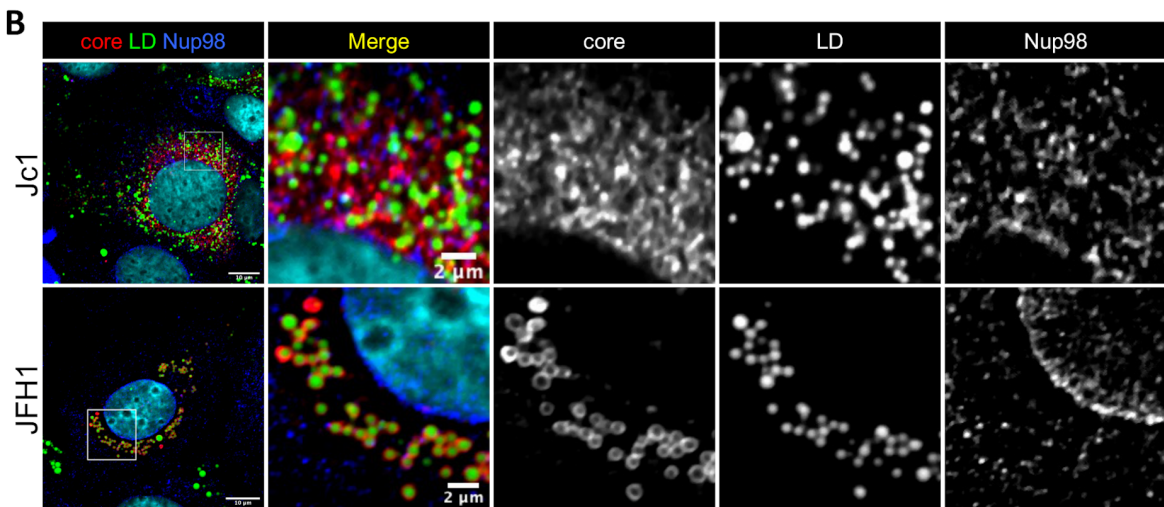

Supplemental Figure 7. Boson et al.

Supplement: FIG S7 [file mbio.02923-21-sf007.pdf]

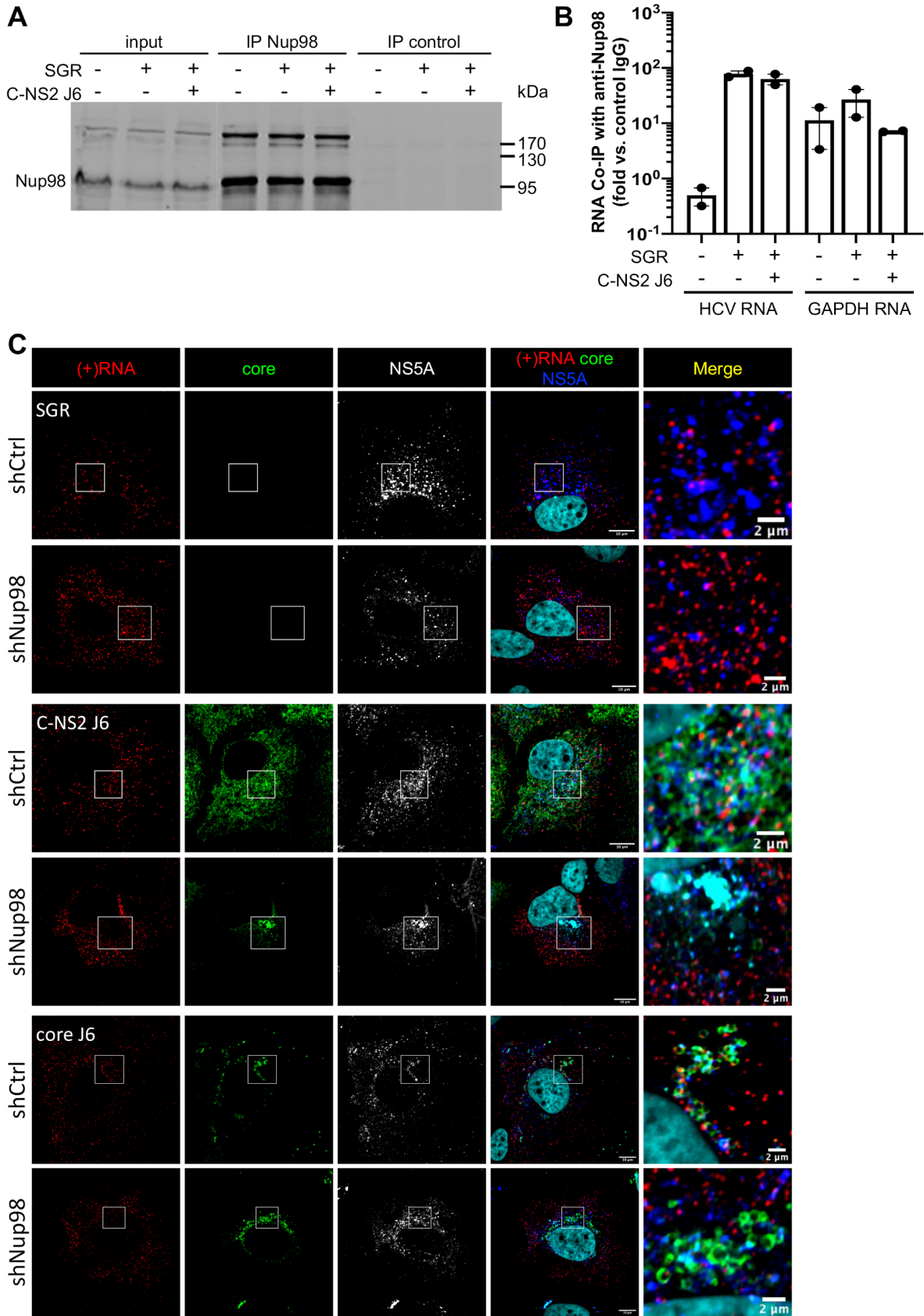

Supplemental Figure 8. Boson et al.

Supplement: FIG S8 [file mbio.02923-21-sf008.pdf]

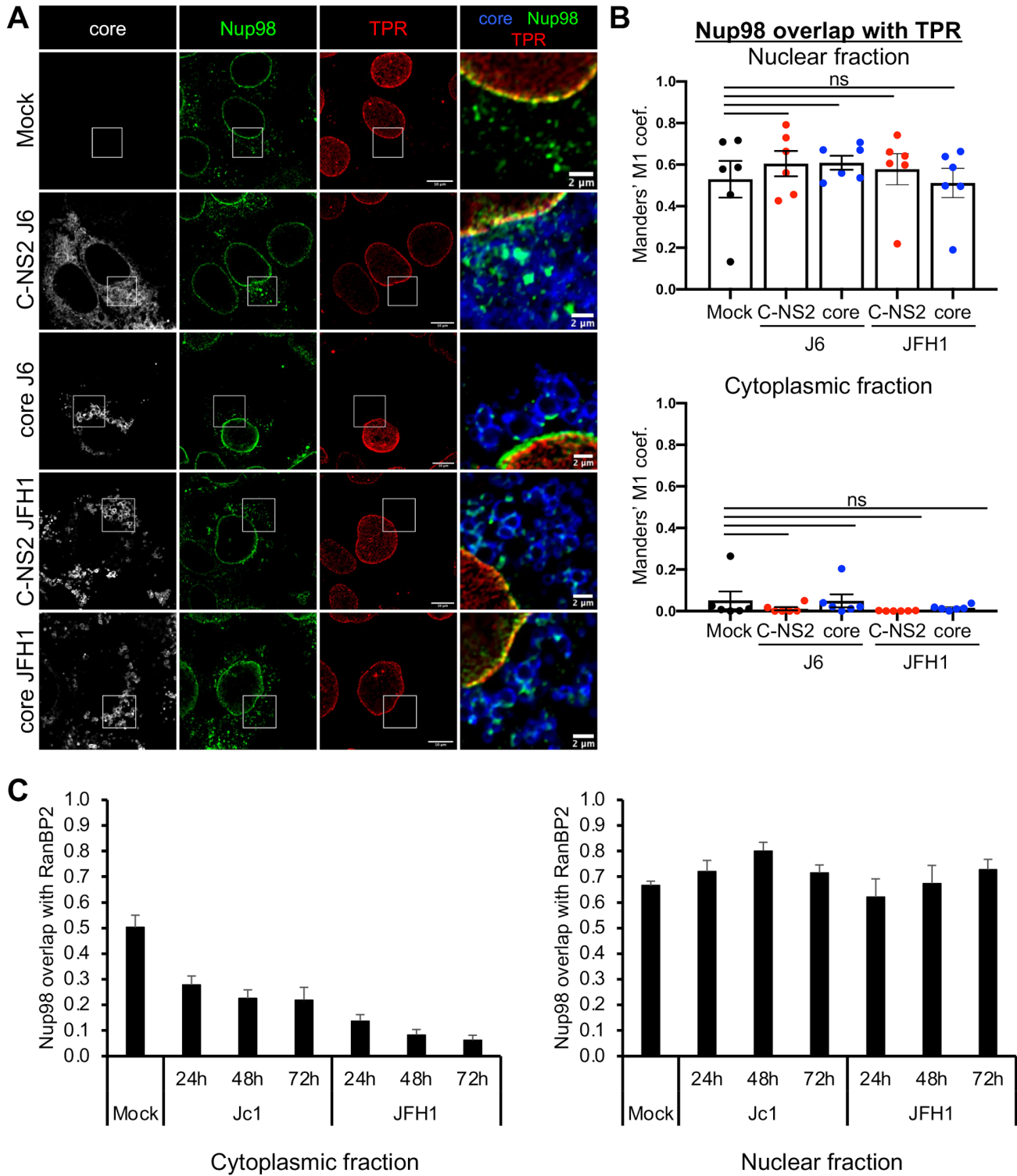

Supplemental Figure 9. Boson et al.

Supplement: FIG S9 [file mbio.02923-21-sf009.pdf]

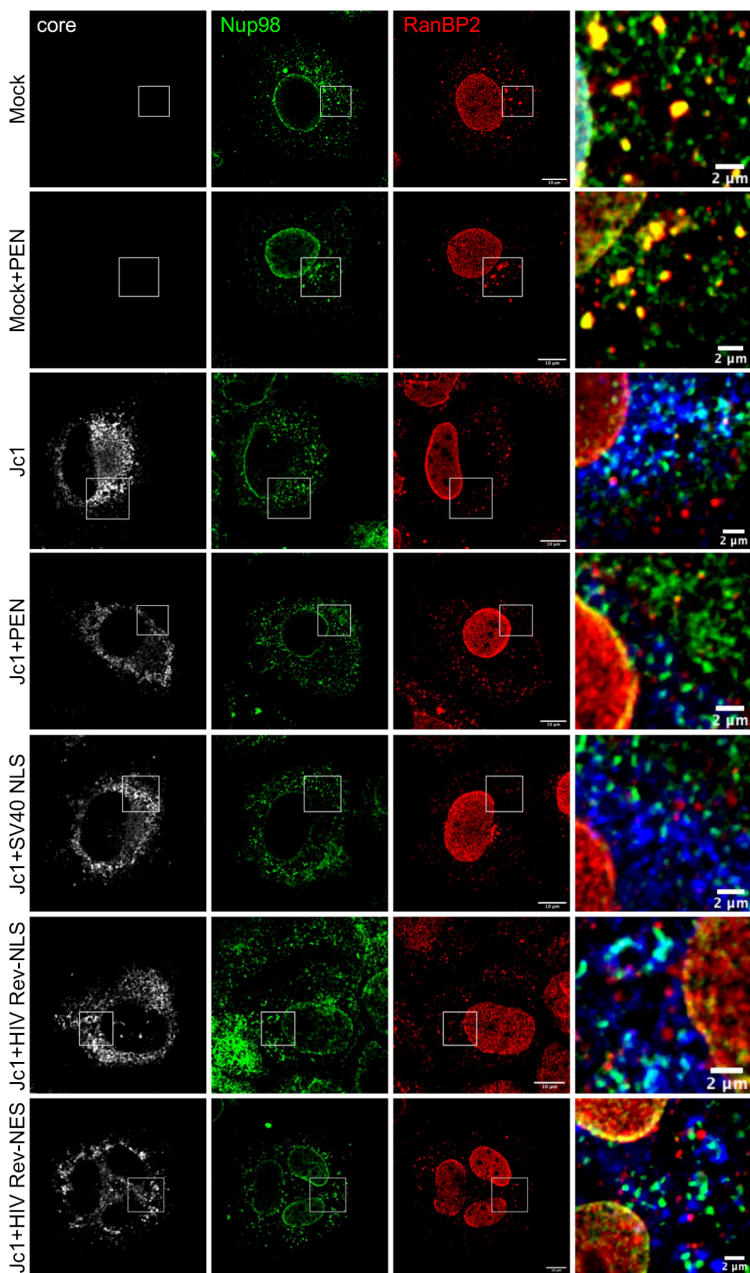

Supplemental Figure 10. Boson et al.

Supplement: FIG S10 [file mbio.02923-21-sf010.pdf]
